# Mendelian randomization identifies causal associations between GWAS-associated bacteria and their metabolites and rheumatoid arthritis

**DOI:** 10.3389/fmicb.2024.1431367

**Published:** 2024-09-02

**Authors:** Donghai Zhou, Wenyue Jiao, Weiman Shi, Qiao Wang, Muzhi Chen

**Affiliations:** ^1^Department of Rheumatology, The Second Affiliated Hospital of Zhejiang Chinese Medical University, Hangzhou, Zhejiang, China; ^2^The Second Clinical Medical College, Zhejiang Chinese Medical University, Hangzhou, Zhejiang, China; ^3^School of Basic Medicine, Zhejiang Chinese Medicine University, Hangzhou, Zhejiang, China

**Keywords:** Mendelian randomization, gut microbiota, metabolites, autoimmune disease, rheumatoid arthritis

## Abstract

**Background:**

Accumulating evidence suggests that an imbalance of gut microbiota is commonly observed in patients with rheumatoid arthritis (RA). However, it remains unclear whether gut microbiota dysbiosis is a cause or consequence of RA, and the mechanisms by which gut dysbiosis contributes to RA have not been fully understood. This study aimed to investigate the causal relationship between gut microbiota and metabolites with RA.

**Methods:**

A two-sample Mendelian randomization analysis was performed to estimate the causality of gut microbiota and metabolites on RA. A genome-wide association study (GWAS) of 211 gut microbiota and 217 metabolites was used as the exposure, whereas RA was treated as the outcome. Inverse variance weighted (IVW) was regarded as the primary approach for calculating causal estimates. MR Egger method, Weighted median method, Simple mode method, and weighted mode method were used for sensitive analysis. Metabolic pathway analysis was performed via the web-based Metaconflict 5.0. Additionally, an animal study was undertaken to evaluate the results inferred by Mendelian randomization.

**Result:**

This study indicated that six gut microbiota taxa (*RuminococcaceaeUCG013, Erysipelotrichia, Erysipelotrichaceae, Erysipelotrichales, Clostridia*, and *Veillonellaceae*) were estimated to exert a positive impact on RA. Conversely, seven gut microbiota taxa (*Oxalobacter, Cyanobacteria, RuminococcaceaeUCG002, LachnospiraceaeUCG010, Christensenellaceae, Oxalobacteraceae, Anaerostipes*) were estimated to exert a negative impact on RA. Three metabolites, namely indole-3-propionate (IPA), glycine and sphingomyelin (SM 16:1), were found to be linked to lower RA risk, while five metabolites (argininosuccinate, CE 20_4, TAG 58_8, PC 40_6, and LPC 20_4) were linked to higher RA risk. Additionally, four metabolic pathways were identified by metabolic pathway analysis. The collagen-induced arthritis (CIA) rats exhibited a higher relative abundance of *Class_Clostridia* and a lower abundance of *Genus_Lachnospiraceae* (*p* < 0.05) than the healthy controls.

**Conclusion:**

This study identified causal associations between specific gut microbiota, metabolites, and RA. These findings support the significant role of gut microbiota and metabolites in RA pathogenesis.

## 1 Introduction

Rheumatoid arthritis (RA) is an autoimmune disease characterized by a symmetrical affliction of the peripheral joints, which leads to swelling, morning stiffness, and persistent joint pain, eventually resulting in disability and posing a global health threat. The global incidence of RA is still high, with an average rate of 1%. Females are more likely to develop RA, especially in their middle years. The pathogenesis of RA remains elusive but may ensue due to intricate interactions between multiple genetic, environmental (including the gut microbiota), and immunological factors (Deane et al., 2017). Therefore, RA remains a challenging condition to treat (Li et al., 2023).

The gastrointestinal (GI) tract contains many beneficial gut microorganisms that are important for regulating immune function and overall health. Imbalances in gut microbiota, known as dysbiosis, have been linked to autoimmune disorders like RA. Studies have shown that RA patients and animal models have dysbiotic gut microbiota compositions, characterized by an imbalance in beneficial and harmful bacteria. This highlights the significance of gut microbiota in the development and progression of RA. Patients with RA showed a higher presence of fecal *Prevotella* spp. during the early stages of the disease, with *Prevotella copri* being particularly prominent and associated with disease progression compared to the control group (Kishikawa et al., 2020). Another case-control study showed that RA patients had a lower diversity index of gut microbiota, a higher abundance of phylum *Verrucomicrobiae* and genus *Akkermansia*, and a lower abundance of *Firmicutes*. Moreover, the alterations in microbiota levels directly correlated with clinical and pathological characteristics like ACPA antibodies, levels of TNF-α and IL-17A, along with immune cell numbers (Chiang et al., 2019; Li Y. et al., 2021). Several animal studies have also demonstrated the critical role of gut microbiota in arthritis development. SKG mice did not develop arthritis under sterile conditions. However, arthritis was induced in SKG mice when they were treated with *Prevotella copri*. In addition, RA models treated with probiotics such as *Lactobacillus rhamnosus GG* showed measurable improvements, including reduced joint swelling (Raaschou et al., 2018).

The relationship between gut dysbiosis and RA remains unclear; it has not been determined whether gut dysbiosis acts as the primary cause or a secondary effect in RA. Moreover, the processes by which gut dysbiosis contributes to RA have yet to be fully understood. Recent studies have indicated that an imbalance in the gut microbiota might lead to RA through the production of functional metabolites, damage to the intestinal mucosal barrier, and molecular mimicry of autoantigens by gut microbes (Yu et al., 2021; He et al., 2022; Zhao et al., 2022). The production of microbial metabolites is recognized as an intermediary mechanism linking gut microbiota and immunity that regulates immune responses and chronic immune disorders such as RA (Yang and Cong, 2021). For example, short-chain fatty acids (SCFAs) synthesized by gut microbiota are noted to engage with germinal center B cells, affect the differentiation of plasmablastes, and play a role in the formation of RA (Rosser et al., 2020).

Moreover, the current knowledge of the microbiome and microbiota-derived metabolites in RA in humans mainly depends on observational studies, which might be susceptible to the effects of confounding factors on the outcomes. In contrast, the Mendelian randomization (MR) approach represents an instrumental variable method that utilizes single-nucleotide polymorphisms (SNPs) as instrumental variables (IVs) to establish causal associations between various traits. MR possesses the distinct advantage of minimizing bias that might arise from confounding variables and reverse causality (Davies et al., 2018). In this study, we employed the two-sample MR method to investigate the possible causal relationship between microbiota, metabolites, and RA using summary statistics from the largest genome-wide association studies (GWAS) thus far.

## 2 Materials and methods

### 2.1 GWAS summary statistics

#### 2.1.1 Gut microbiota

Summary statistics for a gut microbiome were extracted from the MiBioGen Consortium (http://www.mibiogen.org). This GWAS study recruited 18,340 participants from various ethnicities (such as European, American Hispanic/Latin, East Asian, etc.) and ages from 24 cohorts with 211 taxa (i.e., 131 genera, 35 families, 20 orders, 16 classes, and nine phyla). The microbiome GWAS analysis between autosomal human genetic variants and gut microbiome was performed in three ways. Firstly, Spearman's correlation analysis was used to examine the association between SNP dosages and three alpha diversity metrics. Secondly, the loci influencing the covariate-adjusted abundance of bacterial taxa were identified. Thirdly, the loci associated with the probability of the presence vs. absence of the bacterial taxon were identified (Kurilshikov et al., 2021).

### 2.2 Gut metabolites

Summary statistics for gut metabolites were obtained from the Framingham Heart Study Offspring Cohort. This GWAS study recruited 2,076 participants and measured 217 metabolites (113 polar analytes and 104 lipid analytes) in plasma (Rhee et al., 2013).

### 2.3 Rheumatoid arthritis

GWAS summary data for RA were obtained from the IEU GWAS database (https://gwas.mrcieu.ac.uk/datasets/). This GWAS study recruited 19,234 RA patients and 61,565 controls from European and Asian ancestries (Okada et al., 2014).

### 2.4 Instrument variable selection

Firstly, quality control measures were applied to select suitable instrumental variables (IVs). For the gut microbiome data, we aimed to ensure data reliability and an adequate number of SNPs for exposure analysis. After reviewing MR research literature, we set the genome-wide threshold for exposure-associated SNPs at 1.0 × 10^−5^, as widely used in previous MR studies (Sanna et al., 2019; Hu et al., 2024). Regarding SNPs associated with plasma gut metabolites, we applied the standard GWAS thresholds (*p* < 5.0 × 10^−8^). Next, clumping was performed to retain only independent SNPs. Based on previous literature on gut microbiome data analysis (Cai et al., 2022a,b; Guo et al., 2023), the threshold for gut microbiome clumping was set to *r*^2^ < 0.1 and a 500 kb window. For gut metabolite, the threshold was set at *r*^2^ < 0.001 and a window size of 10,000 kb (Hu et al., 2024).

Further, if the outcome datasets lacked SNPs, we selected proxy SNPs following methods from previous studies (Xiang et al., 2021). Using the “extract_outcome_data” function from the TwoSampleMR R package, we identified proxy SNPs with high LD, selecting those with *R*^2^ > 0.8 (Hemani et al., 2018; Hou et al., 2023). To prevent weak instrument bias, the *F*-statistic was utilized to evaluate whether weak IVs were confounded with the selected IVs. IVs with *F*-statistics < 10 were excluded. The equation 2 × EAF × (1–EAF) × beta^2^)/(2 × EAF × (1–EAF) × beta^2^) + (2 × EAF × (1–EAF) × N × SE(beta)^2^ was employed to calculate the *F* statistics for each bacterial taxon, beta^2^/SE^2^ was applied for each SNP extracted from metabolites. EAF denotes effect allele frequency, beta signifies the genetic impact on physical activity, *N* is the GWAS sample size for the SNP-physical activity link, and SE(beta) is the standard error of the genetic effect (Papadimitriou et al., 2020).

### 2.5 Statistical analysis

#### 2.5.1 MR analyses

We performed two-sample MR analysis methods to estimate the causal associations between two instrument exposures (gut microbiota and metabolites) and instrument outcome-RA. Five high-efficiency methods were applied, including the inverse variance weighted (IVW) method, MR Egger method, weighted median method, Simple mode method, and weighted mode method; IVW was regarded as the primary approach. The Wald ratio method was used for the MR analysis for bacterial taxa and metabolites containing only one SNP. Cochrane's *Q*-test and leave-one-out sensitivity analysis were performed to assess the heterogeneity among SNPs. In the presence of heterogeneity (*p* < 0.05), heterogeneity was estimated using the inconsistency index (*I*^2^) value (*I*^2^ values < 50% indicate low to moderate heterogeneity, whereas *I*^2^ ≥ 50% indicate moderate to high heterogeneity) and a random-effects IVW test was utilized. MR-Egger intercept, Mendelian Randomization Pleiotropy RESidual Sum, and MR-PRESSO global test were used to detect the presence of pleiotropy and to eliminate the effects of pleiotropy by removing outliers (Verbanck et al., 2018). The following three conditions were used to determine whether there was a causal effect of human gut microbiome composition or gut metabolite on RA risk: (1) A significant difference in the IVW method (*p* < 0.05), (2) Consistency in the estimation directions of the IVW, weighted median, and MR-Egger methods, and (3) Non-significance in both the MR-Egger intercept test and the MR-PRESSO global test (*p* > 0.05) (Yuan et al., 2022). All analyses were conducted in R Studio (version 4.2.0) using the TwoSampleMR and MRPRESSO packages.

#### 2.5.2 Metabolic pathway analyses

Metabolic pathways were analyzed using the online platform MetaboAnalyst 5.0 (https://www.metaboanalyst.ca/). The Small Molecule Pathway Database (SMPDB) and the Kyoto Encyclopedia of Genes and Genomes (KEGG) database were used in this study, with the significance level set to default.

### 2.6 Animal experiments

#### 2.6.1 Animal model construction

The animal experiments were reviewed and approved by the Ethics Committee of Zhejiang Chinese Medical University (Ref No. IACUU-20190218-01). Twelve specific pathogen-free (SPF) female Wistar rats (6–7 weeks old) were obtained from Shanghai SLAC Laboratory Animal Co., Ltd. After an acclimatization period of 7 days, 12 rats were randomly divided into two groups: (1) control group (CT): injected with 200 μL of 0.9% NaCl solution at the base of the tail on days 0 and 7; (2) collagen-induced arthritis (CIA) group: immunized using bovine Collagen Type II (CII): 200 μg (USA, Chondrex) emulsified with 200 μL incomplete Freund's adjuvant (USA, Chondrex) at the base of the tail on day 0, rats were boosted with 100 μg CII in 100 μL incomplete Freund's adjuvant on day 7 (Rosloniec et al., 2010). On day 21, the rats were sacrificed, and tissues along with fecal samples were collected.

#### 2.6.2 Assessment of arthritis severity

The swelling in the hind paws was measured using a paw volume meter (YSC-7C, China). The ankle joints obtained from rats were immobilized in a solution containing 4% formalin. After fixation, the joints were dehydrated and subsequently embedded in paraffin. The tissue sections were stained with hematoxylin-eosin (H&E), and Safranin O-Fast Green (Saf-O/FG).

#### 2.6.3 Fecal sample storage, DNA extraction, and 16S rRNA sequencing

Following the guidelines provided by the manufacturer, fecal DNA extraction was performed with a kit from Tiangen Biotech Co., Ltd., Beijing, China. We evaluated the DNA's concentration and purity using a NanoDrop 2000 spectrophotometer (Thermo Fisher Scientific, USA). The V3-V4 region of the 16S rRNA gene was amplified using broad-range bacterial primers as previously described (Yu et al., 2018). Following the manufacturer's instructions, we purified, quantified, combined, and sequenced the amplified samples using standard methods and the MiSeq Reagents Kit v3 (600 cycles, Illumina). The genetic sequencing was carried out by Hangzhou Legenomics Bio-Pharm Technology Co., Ltd., based in Zhejiang, China.

Sequences were analyzed using Quantitative Insights into Microbial Ecology (QIIME) (Caporaso et al., 2010). The measurement of Operational Taxonomic Units (OTUs), indicative of the variety of bacterial types, was performed with a threshold of 97% identity for each sample. The abundance of OTUs was established at the genus level (Edgar, 2010), and bacterial taxonomy was determined via the use of the SILVA (Quast et al., 2013) and the NCBI database (Sayers et al., 2019). The OTU table was imported into R software, and alpha and beta diversity metrics were calculated using the “vegan” package. Principal coordinate analysis (PCoA) was employed to examine beta diversity via weighted or unweighted UniFrac analysis utilizing R software.

#### 2.6.4 Statistical analysis

Differences in the relative abundance of gut microorganisms between the CIA and CT groups were analyzed using Student's *t*-tests for data with normal distributions and non-parametric tests for data with non-normal distributions.

## 3 Results

### 3.1 Mendelian randomization

#### 3.1.1 Associations between gut microbiota and RA

**Figure 1:**
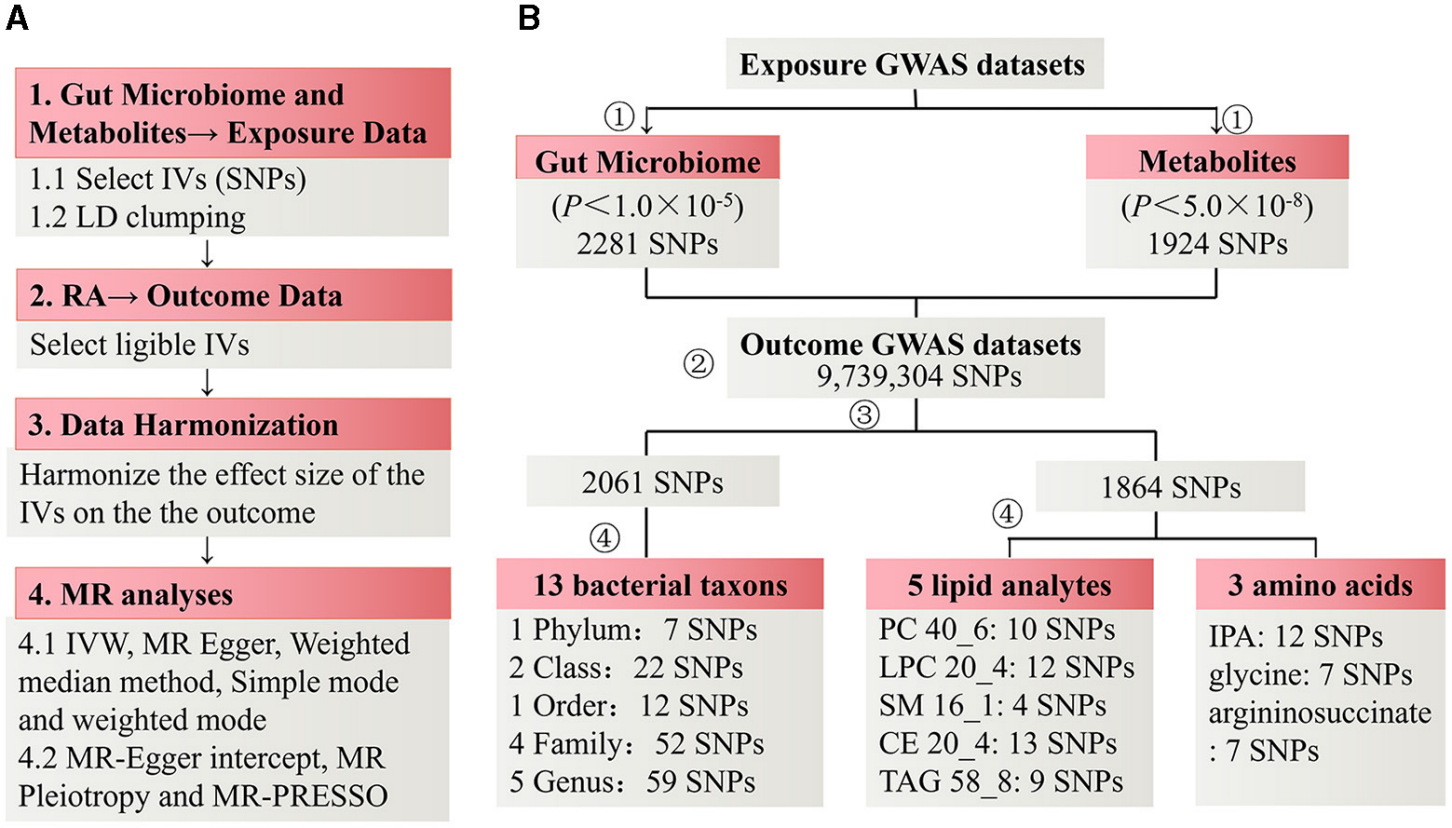
Flowchart of Mendelian randomization analysis.

The Mendelian randomization analysis, illustrated in Figure 1, initially involved 2,281 significant (*p* < 1 × 10^−5^) SNPs or their proxies related to gut microbiota traits as genetic instrumental variables. These variables encompassed 211 gut microbiota traits across multiple taxonomic levels (9 phyla, 16 classes, 20 orders, 36 families, 119 genera). After removing the SNPs with LD effects and independence from RA, 2061 SNPs were selected. The *F*- statistics of each bacterial taxon were all >20, indicating that weak IV bias could be effectively avoided (Supplementary Table 1). The primary data for these SNPs, including their β values, SE, and *p*-values, were systematically compiled for subsequent analyses.

**Figure 2:**
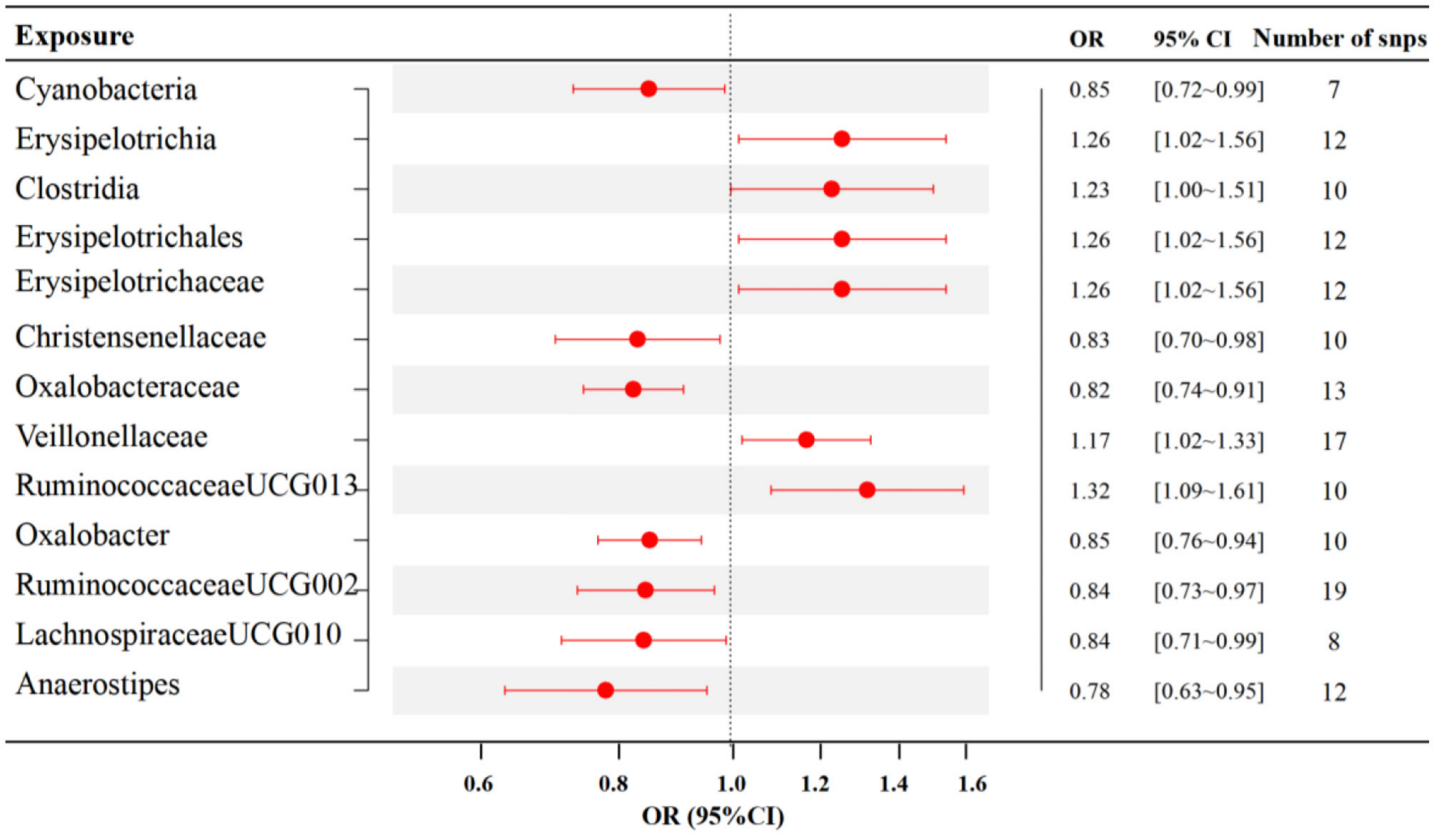
Associations between specific gut microbiota and RA risk (*p* < 1 × 10^−5^). SNP, single nucleotide polymorphism; OR, odds ratio; CI, confidence interval.

The results of IVW analyses demonstrated that *RuminococcaceaeUCG013* [odds ratio (OR) = 1.32, 95% confidence interval (CI), 1.09–1.61, *p* = 0.005], *Erysipelotrichia, Erysipelotrichaceae, Erysipelotrichales* (OR = 1.26, 95% CI, 1.02–1.56, *p* = 0.035), *Clostridia* (OR = 1.23, 95% CI, 1.00–1.51, *p* = 0.049), and *Veillonellaceae* (OR = 1.17, 95% CI, 1.02–1.33, *p* = 0.021) exert a positive impact on RA. *Oxalobacter* (OR =0.85, 95% CI, 0.76–0.94, *p* = 0.002), *Cyanobacteria* (OR = 0.85, 95% CI, 0.72–0.99, *p* = 0.035), *RuminococcaceaeUCG002* (OR = 0.84, 95% CI, 0.73–0.97, *p* = 0.015), *LachnospiraceaeUCG010* (OR = 0.84, 95% CI, 0.71–0.99, *p* = 0.038), *Christensenellaceae* (OR = 0.83, 95% CI, 0.70–0.98, *p* = 0.027), *Oxalobacteraceae* (OR = 0.82, 95% CI, 0.74–0.91, *p* = 0.000), and *Anaerostipes* (OR = 0.78, 95% CI, 0.63–0.95, *p* = 0.016) exerted a negative impact on RA (Figure 2). The estimates of MR Egger, Weighted median, Simple mode, and Weighted mode were consistent with IVW listed in Supplementary Table 1. MR-Egger intercept and Mendelian randomization pleiotropy residual sum and outlier (MR-PRESSO) were utilized to investigate the directional pleiotropy, and all *p*-values exceeded 0.05, suggesting no significant pleiotropy. The Cochran *Q-*statistic of the IVW test and the MR Egger regression were used to assess heterogeneity. No discernible heterogeneity was observed across all studies (Supplementary Table 1).

The findings from the forest plot and scatter plot consistently indicated that *Genus_RuminococcaceaeUCG013* linked to a higher RA risk. Furthermore, the leave-one-out sensitivity test was conducted to assess the individual contribution of a single SNP on the overall analysis, and the results demonstrated that no specific SNP exerted a predominant effect (Figures 3A–C). The scatter plot, forest plot, and leave-one-out plot of the remaining 12 bacterial taxa were presented in Supplementary Figures 1–12.

**Figure 3:**
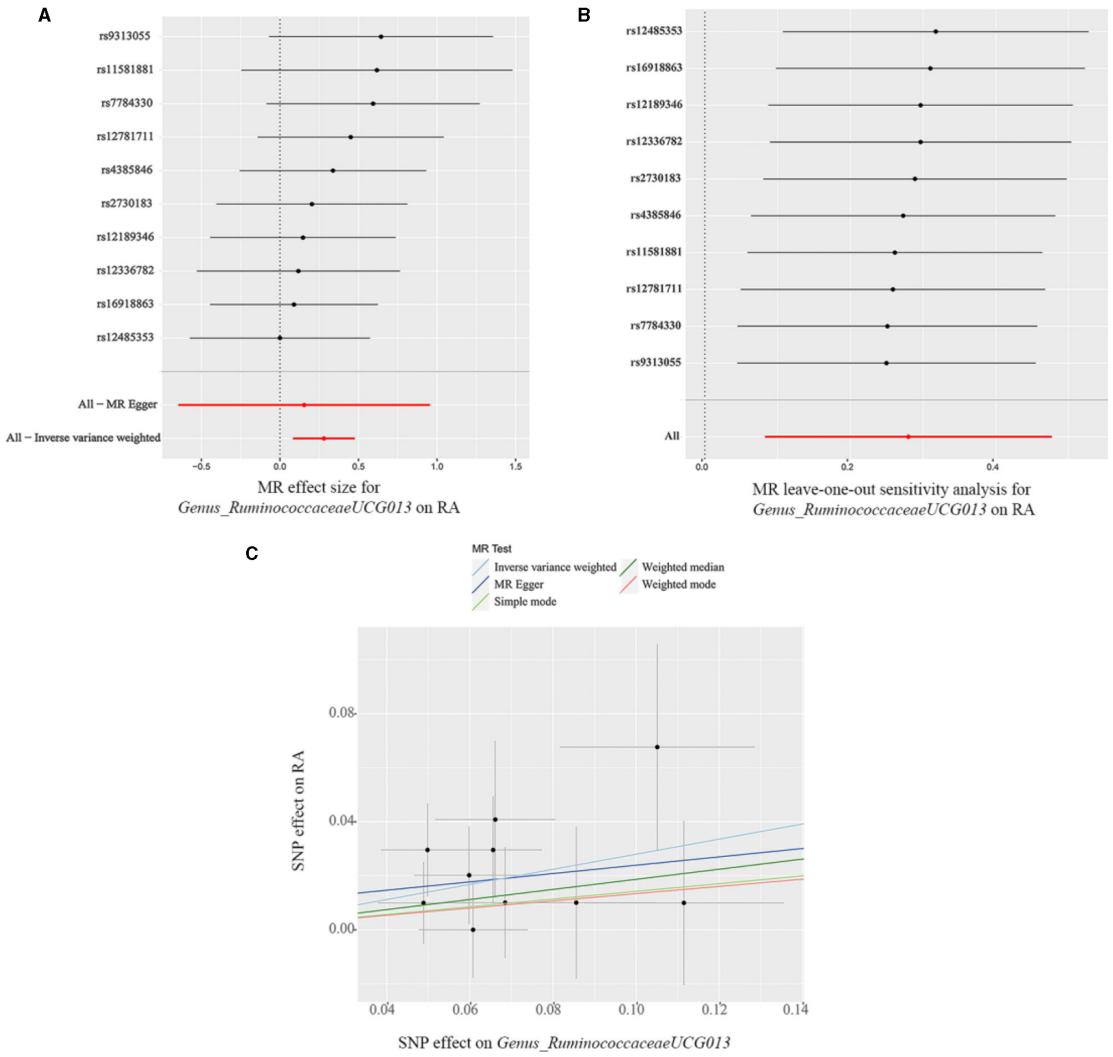
**(A)** Forest plot, **(B)** leave-one-out sensitivity analysis, and **(C)** scatter plot of the causal effect of *Genus_RuminococcaceaeUCG013* on RA risk.

#### 3.1.2 Associations between gut metabolites and RA

We initially included 1,924 significant (*p* < 1 × 10^−5^) SNPs or their proxies that were associated with gut metabolite features as genetic instrumental variables, including 217 metabolites (113 polar analytes and 104 lipid analytes). After removing the SNPs with LD effects and independence from RA, 1,864 SNPs were selected for further analysis. The *F*- statistics of each included SNPs were all >10, indicating that weak IV bias could be effectively avoided. The primary information of SNPs, including β, standard error (SE), and *p*-value, were compiled in Supplementary Table 2.

Twelve gut metabolites were filtered out using the IVW method with *p* < 0.05, in which TAG 60_12 and deoxycholate arginine were excluded because the estimation directions of IVW, weighted median, and MR-Egger methods were inconsistent. The LPC 20_3 gene was removed with significant variability (*I*^2^ ≥ 50%). Furthermore, the results were judged to be reliable, with no evidence of heterogeneity, pleiotropic effects, or outliers, as shown in Supplementary Table 2.

**Figure 4:**
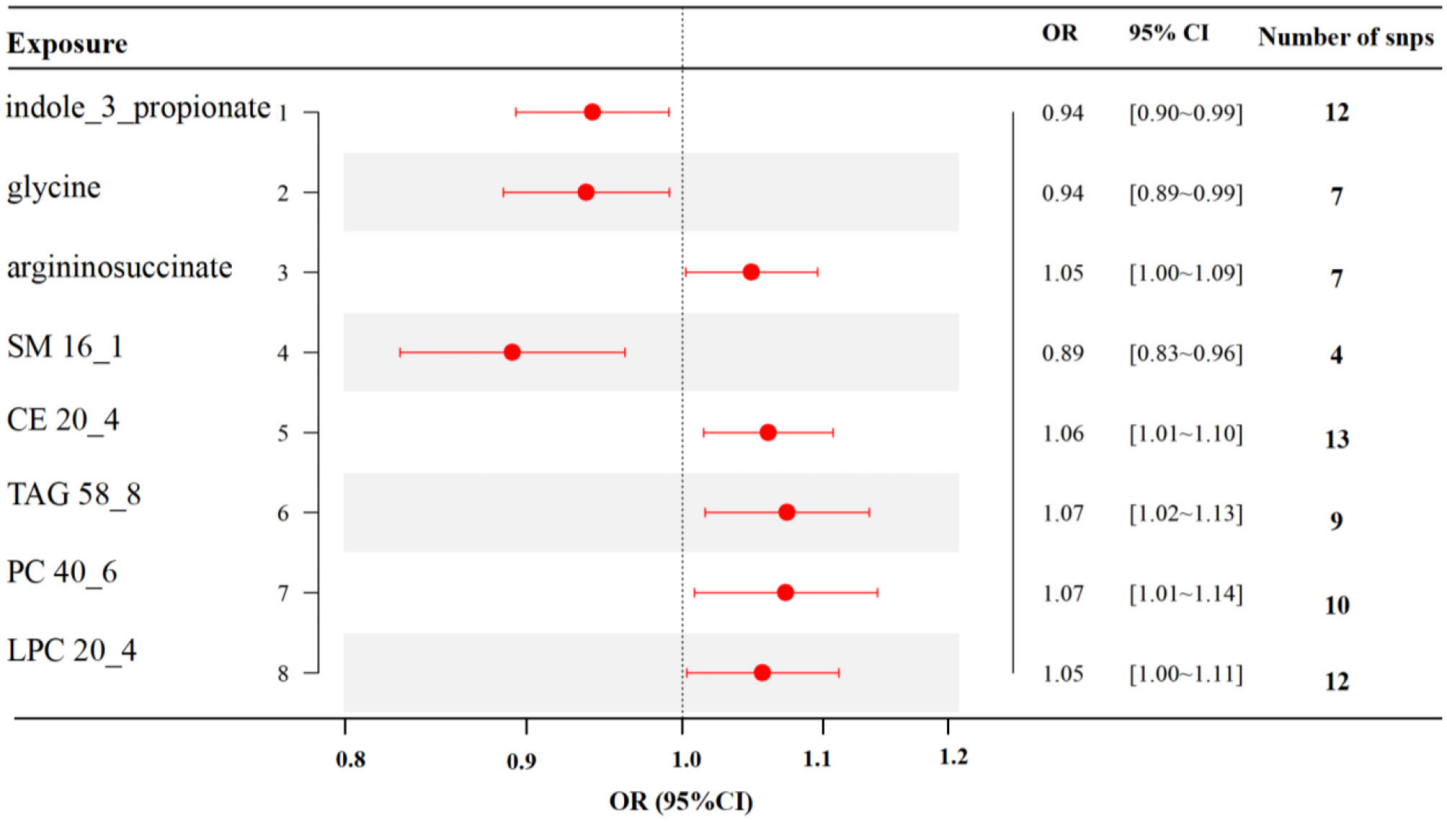
MR results of causal links between gut metabolites and RA risk (*p* < 1 × 10^−5^). SM, sphingomyelin; CE, cholesteryl ester; TAG, triglycerides; PC, phosphatidylcholine; LPC, lysoPC.

MR analyses indicated eight gut metabolites were found to have causal associations with RA, including three amino acids and five lipid analytes (Figure 4). In tryptophan and glycine metabolism, our study revealed that indole-3-propionate (IPA) (OR = 0.94, 95% CI, 0.90–0.99, *p* = 0.021) and glycine (OR = 0.94, 95% CI, 0.89–0.99, *p* = 0.023) were estimated to exert a negative impact on RA. However, argininosuccinate (OR = 1.05, 95% CI, 1.00–1.09, *p* = 0.039) exerted a positive impact on RA. For the lipid analytes, SM 16_1 (OR = 0.89, 95% CI, 0.83–0.96, *p* = 0.003) was estimated to exert a negative impact on RA, CE 20_4 (OR = 1.06, 95% CI, 1.01–1.10, *p* = 0.009), TAG 58_8 (OR = 1.07, 95% CI, 1.02–1.13, *p* = 0.012), PC 40_6(OR = 1.07, 95% CI, 1.01–1.14, *p* = 0.027), and LPC 20_4 (OR = 1.05, 95% CI, 1.00–1.11, *p* = 0.039) were exert a positive impact on RA.

**Figure 5:**
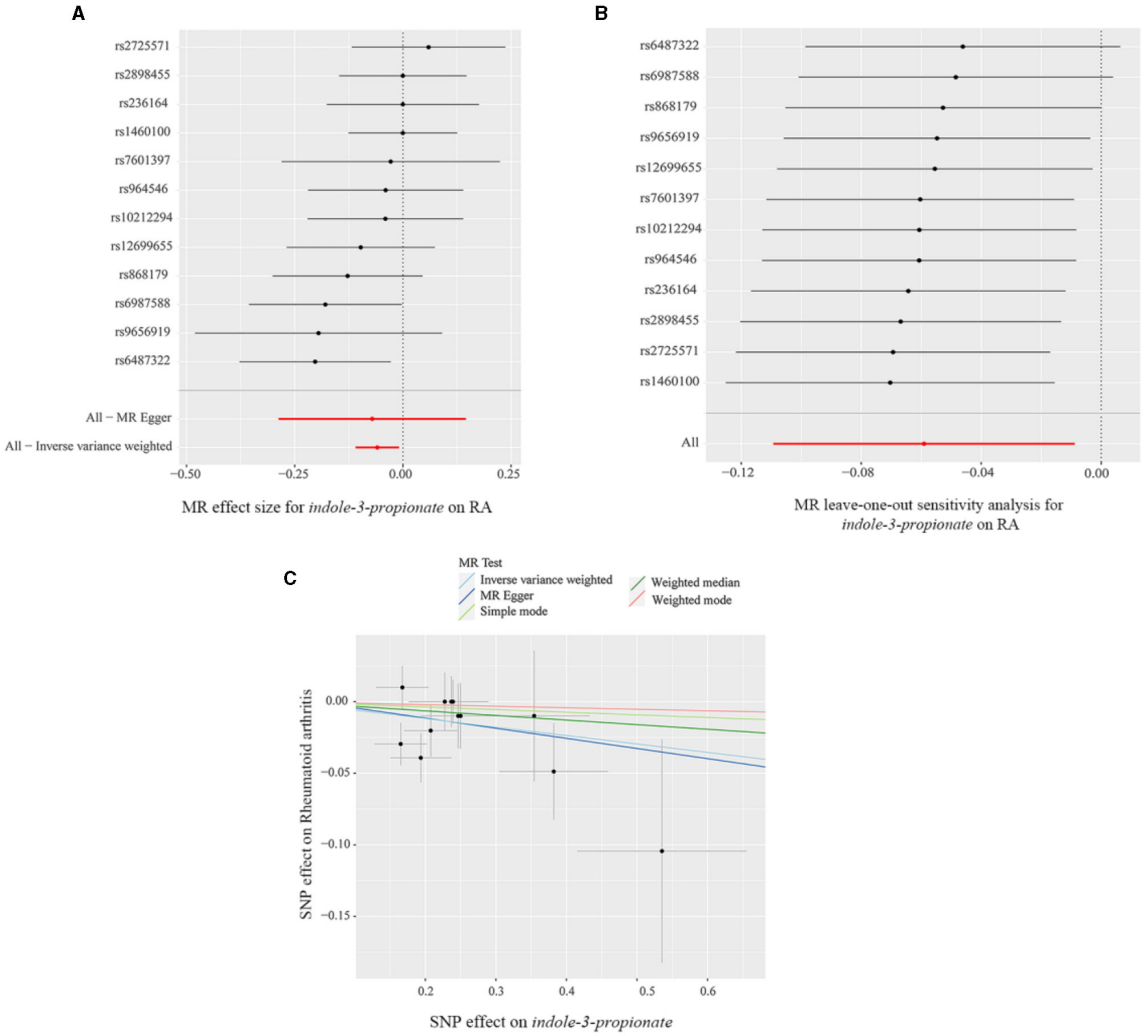
**(A)** Forest plot, **(B)** leave-one-out sensitivity analysis, and **(C)** scatter plot of the causal effect of indole-3-propionate on RA risk.

The scatter plot and forest plot also revealed IPA linked to lower RA risk. Additionally, the leave-one-out sensitivity test showed that no single SNP had a dominant effect on the overall assessment (Figures 5A–C). The scatter plot, forest plot, and leave-one-out plot of the remaining seven gut metabolites were shown in Supplementary Figures 13–19.

#### 3.1.3 Metabolic pathway analysis

**Figure 6:**
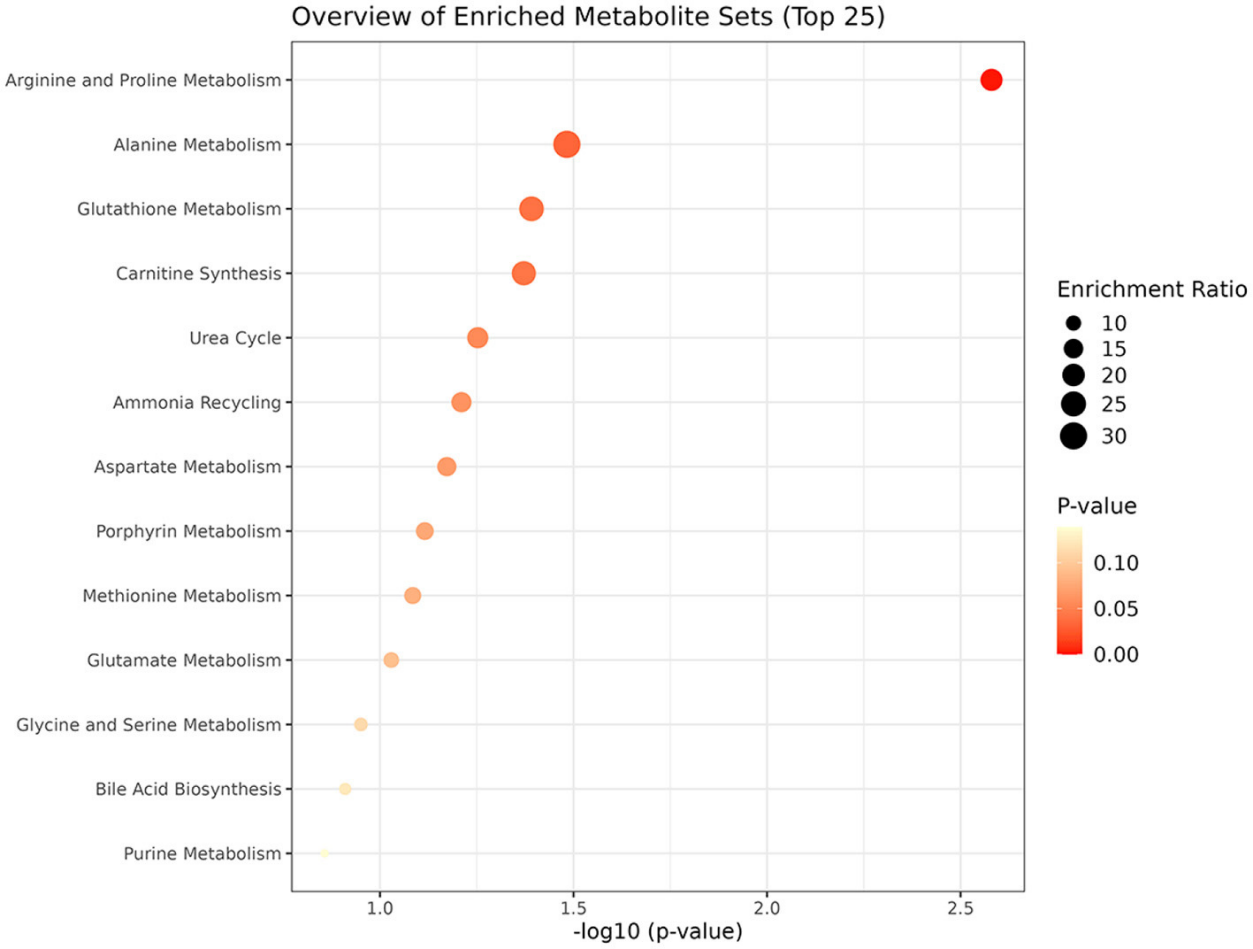
Enriched significant metabolic pathways of RA.

The metabolic pathway analysis of eight metabolites identified four significant metabolic pathways, including Arginine and Proline Metabolism (*p* = 0.00263), Alanine Metabolism (*p* = 0.0329), Glutathione Metabolism (*p* = 0.0406), and Carnitine Synthesis (*p* = 0.0425), as shown in Figure 6 and Supplementary Table 3.

### 3.2 Animal experiments

#### 3.2.1 Assessment of arthritis severity

The CIA group exhibited significant joint swelling (*p* < 0.05). Pathologically, there was notable destruction of the joint structure, narrowing of the joint space, and extensive infiltration of inflammatory cells in the CIA group (Supplementary Figures 20A, B). These results suggest that a CIA rat model was effectively established.

### 3.3 β-diversity

**Figure 7:**
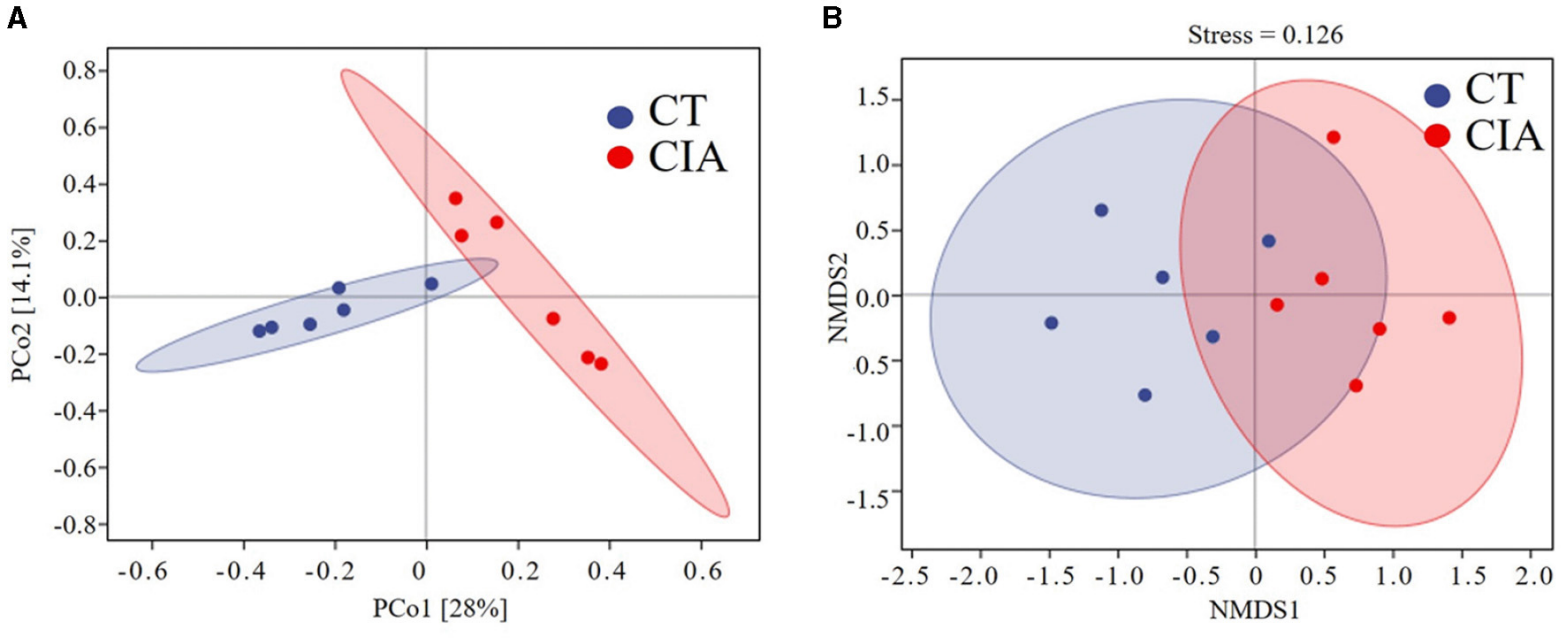
**(A)** β-diversity was described by PCoA based on weighted UniFrac distance metric; **(B)** and non-metric multidimensional scaling (NMDS) analysis. CIA, collagen-induced arthritis group; CT, healthy control group.

We performed a principal component analysis (PCoA) based on the weighted UniFrac distance and NMDS analysis to assess β-diversity. The PCoA analysis indicated that the two groups achieved statistical significance (*p* = 0.004) (Figure 7A) and the NMDS analysis (Stress = 0.126, *p* = 0.004). The results were consistent with the previous findings (Figure 7B).

#### 3.3.1 Validation of MR results

**Figure 8:**
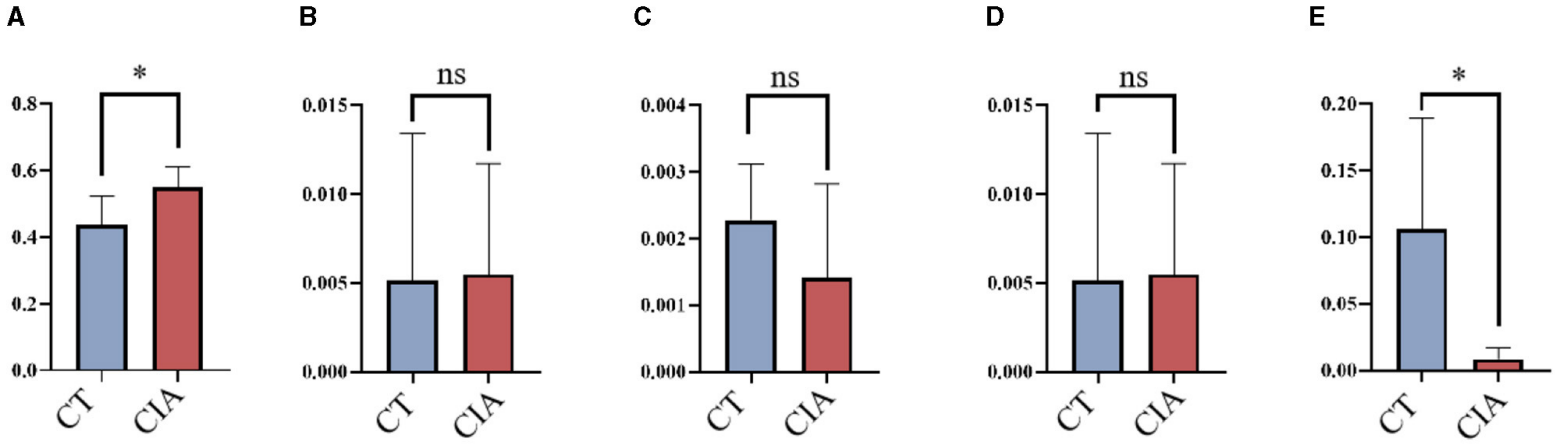
The relative abundance of bacterial taxa by bar plots **(A–E)** the relative abundance of *Class_Clostridia, Order_Erysipelotrichales, Family_Christensenellaceae, Family_Erysipelotrichaceae*, and *Genus_Lachnospiraceae* in the control group and CIA group. *p < 0.05. CIA, collagen-induced arthritis group; CT, healthy control group.

To evaluate the MR findings, animal experiments were performed. We extracted the relative abundance of *Class_Clostridia, Order_Erysipelotrichales, Family_Christensenellaceae, Family_Erysipelotrichaceae*, and *Genus_Lachnospiraceae* and compared them using bar plots (Figures 8A–E).

## 4 Discussion

Our study showed that increased abundance of *RuminococcaceaeUCG013, Erysipelotrichia, Erysipelotrichaceae, Erysipelotrichales, Clostridia*, and *Veillonellaceae* was linked to higher RA risk. Conversely, *Cyanobacteria, RuminococcaceaeUCG002, Oxalobacter, LachnospiraceaeUCG010, Christensenellaceae, Oxalobacteraceae*, and *Anaerostipes* were estimated to exert a negative impact on RA. Furthermore, we identified three amino acids and five lipid analytes, which are linked to the gut microbiota, as having a causal relationship with RA risk, highlighting their importance in the gut microbiota-metabolite axis.

The GI tract is abundantly colonized with trillions of symbiotic gut microbiomes that participate in the modulation and maintenance of the host immune system (Zhao et al., 2022). Altering specific bacterial lineages may result in dysbiosis within the gut microbiota, ultimately leading to the onset of autoimmune diseases (Wang et al., 2022). Multiple studies have shown that the microbiota composition, including pathogenic bacteria and probiotics, is significantly altered in RA patients. Probiotics, such as *Bifidobacterium*, are live microorganisms that can benefit health by competing with pathogenic bacteria for nutrition and colonization sites (Chen et al., 2021). Probiotics have been shown to reduce RA symptoms by producing antibiotics and strengthening the intestinal barrier, which enhances immune function modulation (Ferro et al., 2021). However, the causal relationship between RA risk and gut microbiota dysbiosis is ambiguous. This study is the first MR analysis that explores this topic from phylum to genus levels of 211 taxa. Seven bacterial taxa were estimated to exert a negative impact on RA. Among these, *Lachnospiraceae* and *Ruminococcaceae* are recognized as probiotic strains that colonize the human gut. Takahashi reported the abundance of *Lachnospiraceae* was downregulated in RA patients compared to healthy controls. Further animal experiments have identified *Lachnospiraceae* as one of the major butyrate producers that can limit the autoimmune response by promoting the differentiation of follicular regulatory T cells (Takahashi et al., 2020). Data from two cohorts identified that RA patients had less *Firmicutes* of the *Ruminococcaceae* family and more *Proteobacteria* of the *Enterobacteriaceae* family than HC, indicating the protective effect of *Ruminococcaceae* on RA (Breban et al., 2017). Interestingly, our analysis showed that the genera *RuminococcaceaeUCG002* and *RuminococcaceaeUCG013* have varying impacts on RA risk, which reminds us that inconsistencies in previous clinical studies may be due to insufficiently digging deeper into the classification at the genera level of gut microbiota. A few studies have reported the role of the family *Oxalobacteraceae* and genus *Oxalobacter* in RA. A case-control study reported that the relative abundance of *Oxalobacter* was negatively related to TNF-α, IL-6, and IL-17 in RA patients. These cytokines are closely linked to bone erosion via enhanced differentiation and activation of osteoclasts (Li Y. et al., 2021). *Cyanobacteria, Christensenellaceae*, and *Anaerostipes* have not been reported to be protective factors for RA, while alterations in other autoimmune diseases have been reported. For example, Ma et al. (2022) reported that Anaerostipes was enriched in healthy controls compared to Crohn's patients. We also identified six bacterial taxa that were estimated to exert a positive impact on RA. The family *Erysipelotrichaceae* and the class *Clostridia* are proinflammatory bacteria overrepresented in RA animal models (Nguyen et al., 2022; Wang et al., 2023). The results of *Veillonellaceae* in this study were also consistent with the previous literature (Manoil et al., 2021). Regarding class *Erysipelotrichia* and order *Erysipelotrichales*, there are few reports about their role in RA. Overall, these results suggest that dysregulation of gut microbiota might contribute to the development of RA, though the underlying mechanisms are still to be elucidated.

Eight gut metabolites were identified to have causal associations from this MR analysis: IPA, glycine, argininosuccinate, SM 16_1, CE 20_4, TAG 58_8, PC 40_6, and LPC 20_4. Among these, the metabolic pathway analysis highlighted four pathways causally associated to RA, some of which are well-documented in experimental studies for their role in RA pathogenesis. For instance, recent integrated analyses of plasma metabolomics, intestinal bacteria, and fungi have shown that arginine and proline metabolic pathways are involved in RA pathogenesis (Zhu et al., 2022).

**Figure 9:**
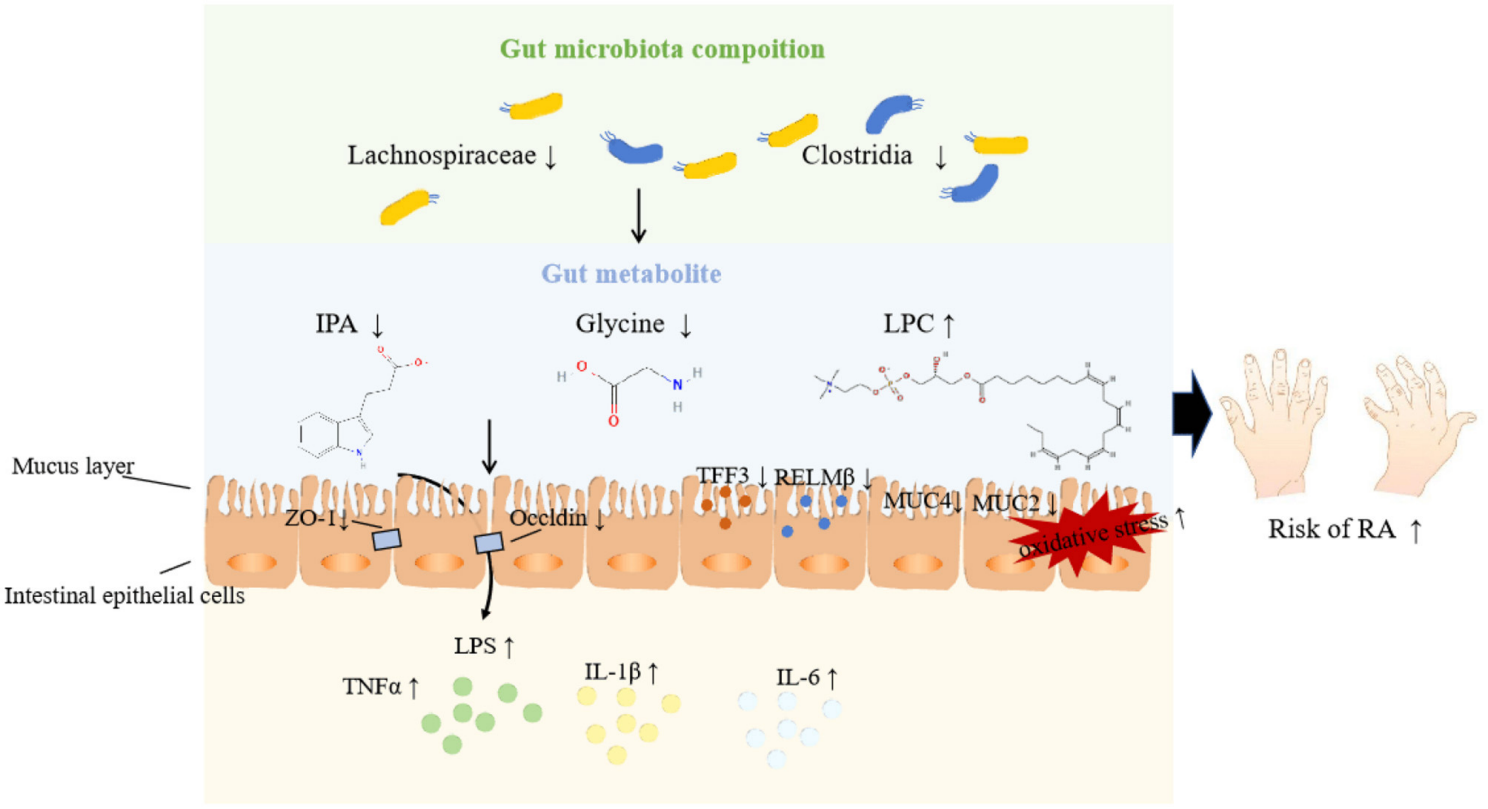
Potential pathways underpinning the relationship among gut microbiota, metabolites, and RA.

Among these eight metabolites, IPA is a tryptophan-derived intestinal bacterial metabolite known for inducing the expression of tight junction proteins such as ZO-1 and Occludin, promoting intestinal epithelium homeostasis, inhibiting NF-κB signaling, and reducing levels of proinflammatory cytokines like TNF-α, IL-1β, and IL-6 (Zhao et al., 2019). Additionally, IPA enhances the protective mucus barrier by increasing the production of mucins, specifically MUC2 and MUC4, and secretion products from goblet cells, including TFF3 and RELMβ (Li J. et al., 2021). Its production is closely linked to the presence of the genus *Lachnospiraceae*, known for its protective effects against RA. Thus, we speculate that probiotic bacteria may metabolize IPA to bolster intestinal barrier functions, thereby mitigating the inflammatory responses associated with RA injury, as illustrated in Figure 9. Similar mechanisms have been observed in other diseases (Zhao et al., 2019; Serger et al., 2022). Supporting our findings, other metabolites like LPC 20_4 and argininosuccinate are implicated in RA pathogenesis, while glycine appears to exert a protective effect by reducing oxidative stress on intestinal epithelial cells, decreasing pro-inflammatory cytokine release, and alleviating epithelial barrier damage (Marchesini et al., 2003; Tang et al., 2021). However, research on the roles of remaining metabolites (SM 16_1, CE 20_4, TAG 58_8, PC 40_6) in RA remains limited.

The primary strength of this study lies in the application of the MR approach, which minimizes both confounding and reverse causality, providing a more robust assessment of causality than observational studies. To our knowledge, this is the first MR analysis examining the causal relationships between gut microbiome, metabolites, and RA. Various analyses were conducted to assess the robustness of the results. Moreover, animal experiments were employed to evaluate the outcomes of the MR analysis, reinforcing the conviction of the causal associations between gut microbiota and RA. However, our study has some limitations. Firstly, due to the multi-stage statistical approach, the adjustment for FDR was not conducted to identify potential strains and metabolites that may be causally associated with rheumatoid arthritis to the greatest extent possible. Secondly, our animal experiments do not definitively establish a causal association between gut microbiota and RA; further gut microbiota transplantation studies are needed to clarify this association. Thirdly, gut microbiota composition is influenced by various factors such as diet, lifestyle, gender, sex hormones, disease severity, and medication status. However, further subgroup analyses were challenging to perform due to the unavailability of detailed information.

## 5 Conclusion

Using MR, our study identified causal associations between specific gut microbiota, metabolites, and RA. These findings support the significant role of gut microbiota and metabolites in RA pathogenesis, particularly highlighting the *Genus_Lachnospiraceae* and IPA.

## Data Availability

The original contributions presented in the study are included in the article/Supplementary material, further inquiries can be directed to the corresponding authors.
