# Supplementary material for: Mendelian randomization identifies causal associations between GWAS-associated bacteria and their metabolites and rheumatoid arthritis
Source: Front Microbiol. 2024 Sep 2;15:1431367. doi: 10.3389/fmicb.2024.1431367 (PMC11404690; doi:10.3389/fmicb.2024.1431367)
Supplement: Supplementary file 1 [file Data_Sheet_1.PDF]

Supplementary Table 1: MR results of causal links between gut microbiome and RA risk ($p < 1 \times 10^{-5}$).

	Classification	Nsnp	Methods	β	SE	OR(95% CI)	*p* value	Heterogeneity		Horizontal pleiotropy			MR PRESSO	F statistic
								Cochran's Q	*p* value	Egger intercept	SE	*p* value	*p* value	
Phylum	Cyanobacteria	7	Inverse variance weighted	-0.167	0.079	0.846	0.035	6.325	0.388	0.005	0.033	0.875	0.416	27.892
			MR Egger	-0.212	0.288	0.809	0.495	6.291	0.279					
			Weighted median	-0.162	0.099	0.850	0.101							
			Simple mode	-0.320	0.172	0.726	0.113							
			Weighted mode	-0.279	0.178	0.757	0.168							
Class	Erysipelotrichia	12	Inverse variance weighted	0.229	0.108	1.257	0.035	14.147	0.225	0.010	0.034	0.773	0.244	22.610
			MR Egger	0.059	0.584	1.061	0.921	14.024	0.172					
			Weighted median	0.358	0.130	1.430	0.006							
			Simple mode	0.429	0.221	1.536	0.078							
			Weighted mode	0.441	0.212	1.554	0.062							
	Clostridia	10	Inverse variance weighted	0.208	0.106	1.231	0.049	7.489	0.586	-0.001	0.034	0.967	0.635	23.301
			MR Egger	0.230	0.539	1.259	0.681	7.487	0.485					
			Weighted median	0.147	0.148	1.159	0.321							

			Simple mode	0.108	0.238	1.114	0.662							
			Weighted mode	0.115	0.222	1.122	0.616							
Order	Erysipelotrichales	12	Inverse variance weighted	0.229	0.108	1.257	0.035	14.147	0.225	0.010	0.034	0.773	0.244	22.610
			MR Egger	0.059	0.584	1.061	0.921	14.024	0.172					
			Weighted median	0.358	0.138	1.430	0.010							
			Simple mode	0.429	0.230	1.536	0.089							
			Weighted mode	0.441	0.235	1.554	0.088							
Family	Erysipelotrichaceae	12	Inverse variance weighted	0.229	0.108	1.257	0.035	14.147	0.225	0.010	0.034	0.773	0.244	22.610
			MR Egger	0.059	0.584	1.061	0.921	14.024	0.172					
			Weighted median	0.358	0.138	1.430	0.010							
			Simple mode	0.429	0.230	1.536	0.089							
			Weighted mode	0.441	0.235	1.554	0.088							
	Christensenellaceae	10	Inverse variance weighted	-0.190	0.086	0.827	0.027	5.082	0.827	0.017	0.015	0.281	0.865	22.772
			MR Egger	-0.385	0.189	0.680	0.076	3.743	0.880					
			Weighted median	-0.221	0.121	0.801	0.067							
			Simple mode	-0.010	0.183	0.990	0.958							
			Weighted mode	-0.239	0.131	0.788	0.101							

	Oxalobacteraceae	13	Inverse variance weighted	-0.199	0.052	0.820	0.000		6.712	0.876	-0.005	0.030	0.865	0.927	24.400
			MR Egger	-0.159	0.235	0.853	0.514		6.682	0.824					
			Weighted median	-0.161	0.071	0.851	0.023								
			Simple mode	-0.205	0.108	0.815	0.082								
			Weighted mode	-0.185	0.101	0.831	0.092								
	Veillonellaceae	17	Inverse variance weighted	0.155	0.067	1.168	0.021		6.594	0.980	-0.002	0.011	0.870	0.987	28.448
			MR Egger	0.178	0.152	1.195	0.258		6.567	0.969					
			Weighted median	0.194	0.097	1.214	0.046								
			Simple mode	0.218	0.145	1.244	0.153								
			Weighted mode	0.207	0.111	1.229	0.082								
Genus	RuminococcaceaeUCG013	10	Inverse variance weighted	0.280	0.101	1.323	0.005		4.668	0.862	0.008	0.027	0.759	0.891	22.478
			MR Egger	0.154	0.408	1.167	0.715		4.567	0.803					
			Weighted median	0.187	0.131	1.206	0.153								
			Simple mode	0.142	0.211	1.153	0.517								
			Weighted mode	0.134	0.194	1.143	0.507								
	Oxalobacter	10	Inverse variance weighted	-0.165	0.054	0.848	0.002		4.928	0.841	-0.007	0.041	0.868	0.863	25.797
			MR Egger	-0.117	0.288	0.890	0.696		4.899	0.768					

		Weighted median	-0.151	0.073	0.860	0.038							
		Simple mode	-0.181	0.121	0.834	0.170							
		Weighted mode	-0.184	0.114	0.832	0.139							
RuminococcaceaeUCG002	19	Inverse variance weighted	-0.173	0.072	0.841	0.015	20.115	0.326	0.031	0.015	0.056	0.387	24.664
		MR Egger	-0.586	0.213	0.556	0.013	15.920	0.530					
		Weighted median	-0.191	0.102	0.826	0.062							
		Simple mode	-0.138	0.174	0.871	0.437							
		Weighted mode	-0.189	0.125	0.828	0.149							
LachnospiraceaeUCG010	8	Inverse variance weighted	-0.178	0.086	0.837	0.038	0.963	0.995	-0.011	0.016	0.540	0.997	26.440
		MR Egger	-0.065	0.194	0.937	0.750	0.540	0.997					
		Weighted median	-0.142	0.115	0.868	0.219							
		Simple mode	-0.138	0.148	0.871	0.382							
		Weighted mode	-0.133	0.137	0.875	0.362							
Anaerostipes	12	Inverse variance weighted	-0.255	0.106	0.775	0.016	12.583	0.321	0.010	0.027	0.711	0.341	29.428
		MR Egger	-0.413	0.428	0.662	0.358	12.402	0.259					
		Weighted median	-0.193	0.143	0.824	0.176							
		Simple mode	-0.253	0.274	0.777	0.376							

Weighted mode	-0.257	0.264	0.773	0.350

Nsnp: number of SNPs; SE: standard error; OR:odds ratio.

Supplementary Table 2: MR results of causal links between gut metabolites and RA risk ($p < 5 \times 10^{-8}$)

Metabolites	Nsnp	Methods	β	SE	OR (95% CI)	*p* value	Heterogeneity		Horizontal pleiotropy			MR PRESSO	F statistic
							Cochran's Q	*p* value	Egger intercept	SE	*p* value	*p* value	
SM 16_1	4	Inverse variance weighted	-0.1116	0.0377	0.8944	0.0030	2.1279	0.5463	0.0186	0.0336	0.6354	0.6220	22.4138
		MR Egger	-0.1751	0.1207	0.8394	0.2800	1.8213	0.4023					
		Weighted median	-0.0951	0.0497	0.9092	0.0600							
		Simple mode	-0.0906	0.0748	0.9134	0.3100							
		Weighted mode	-0.0890	0.0486	0.9149	0.1600							
CE 20_4	13	Inverse variance weighted	0.0567	0.0217	1.0583	0.0090	18.5551	0.0998	-0.0219	0.0153	0.1789	0.1260	39.8297
		MR Egger	0.1202	0.0489	1.1277	0.0318	15.6265	0.1556					
		Weighted median	0.0604	0.0244	1.0622	0.0134							
		Simple mode	0.0007	0.0454	1.0007	0.9882							
		Weighted mode	0.0740	0.0260	1.0768	0.0147							

TAG 58_8	9	Inverse variance weighted	0.0690	0.0275	1.0714	0.0120	4.2730	0.8317	0.0018	0.0203	0.9314	0.8510	26.0998
		MR Egger	0.0623	0.0796	1.0643	0.4592	4.2650	0.7488					
		Weighted median	0.0609	0.0365	1.0628	0.0954							
		Simple mode	0.0597	0.0556	1.0616	0.3142							
		Weighted mode	0.0579	0.0507	1.0596	0.2869							
indole_3_propionate	12	Inverse variance weighted	-0.0591	0.0256	0.9427	0.0210	9.7933	0.5491	0.0027	0.0247	0.9149	0.5600	24.6226
		MR Egger	-0.0708	0.1104	0.9316	0.5357	9.7813	0.4599					
		Weighted median	-0.0321	0.0373	0.9684	0.3894							
		Simple mode	-0.0184	0.0609	0.9818	0.7682							
		Weighted mode	-0.0106	0.0499	0.9895	0.8361							
glycine	7	Inverse variance weighted	-0.0631	0.0278	0.9388	0.0230	3.9244	0.6869	0.0072	0.0186	0.7166	0.7340	54.3542
		MR Egger	-0.0856	0.0648	0.9180	0.2436	3.7767	0.5820					
		Weighted median	-0.0532	0.0330	0.9482	0.1063							
		Simple mode	0.0000	0.0556	1.0000	1.0000							
		Weighted mode	-0.0767	0.0380	0.9261	0.0900							
PC 40_6	10	Inverse variance weighted	0.0680	0.0307	1.0704	0.0270	14.8426	0.0954	0.0012	0.0218	0.9562	0.1020	26.8395
		MR Egger	0.0633	0.0889	1.0654	0.4965	14.8366	0.0624					

		Weighted median	0.0510	0.0372	1.0523	0.1708							
		Simple mode	0.0590	0.0741	1.0608	0.4460							
		Weighted mode	0.0386	0.0513	1.0394	0.4707							
LPC 20_4	12	Inverse variance weighted	0.0528	0.0255	1.0542	0.0390	16.9193	0.1103	-0.0098	0.0173	0.5842	0.1670	41.1270
		MR Egger	0.0841	0.0614	1.0878	0.2004	16.3950	0.0889					
		Weighted median	0.0747	0.0264	1.0776	0.0047							
		Simple mode	0.0326	0.0583	1.0332	0.5869							
		Weighted mode	0.0802	0.0261	1.0835	0.0106							
argininosuccinate	7	Inverse variance weighted	0.0455	0.0221	1.0466	0.0390	2.8969	0.8217	0.0085	0.0225	0.7223	0.8310	23.2372
		MR Egger	0.0242	0.0609	1.0245	0.7079	2.7555	0.7376					
		Weighted median	0.0539	0.0295	1.0554	0.0678							
		Simple mode	0.0627	0.0432	1.0647	0.1964							
		Weighted mode	0.0693	0.0377	1.0718	0.1156							

Nsnp: number of SNPs; SE: standard error; OR:odds ratio.

Supplementary Table 3: Enriched significant metabolic pathways of RA (p <0.05)

metabolic pathways	p value	Holm p	FDR
Arginine and Proline Metabolism	0.00263	0.258	0.258
Alanine Metabolism	0.0329	1	0.896
Glutathione Metabolism	0.0406	1	0.896
Carnitine Synthesis	0.0425	1	0.896

Holm p : Holm-Bonferroni Adjusted p-value; FDR: False Discovery Rate

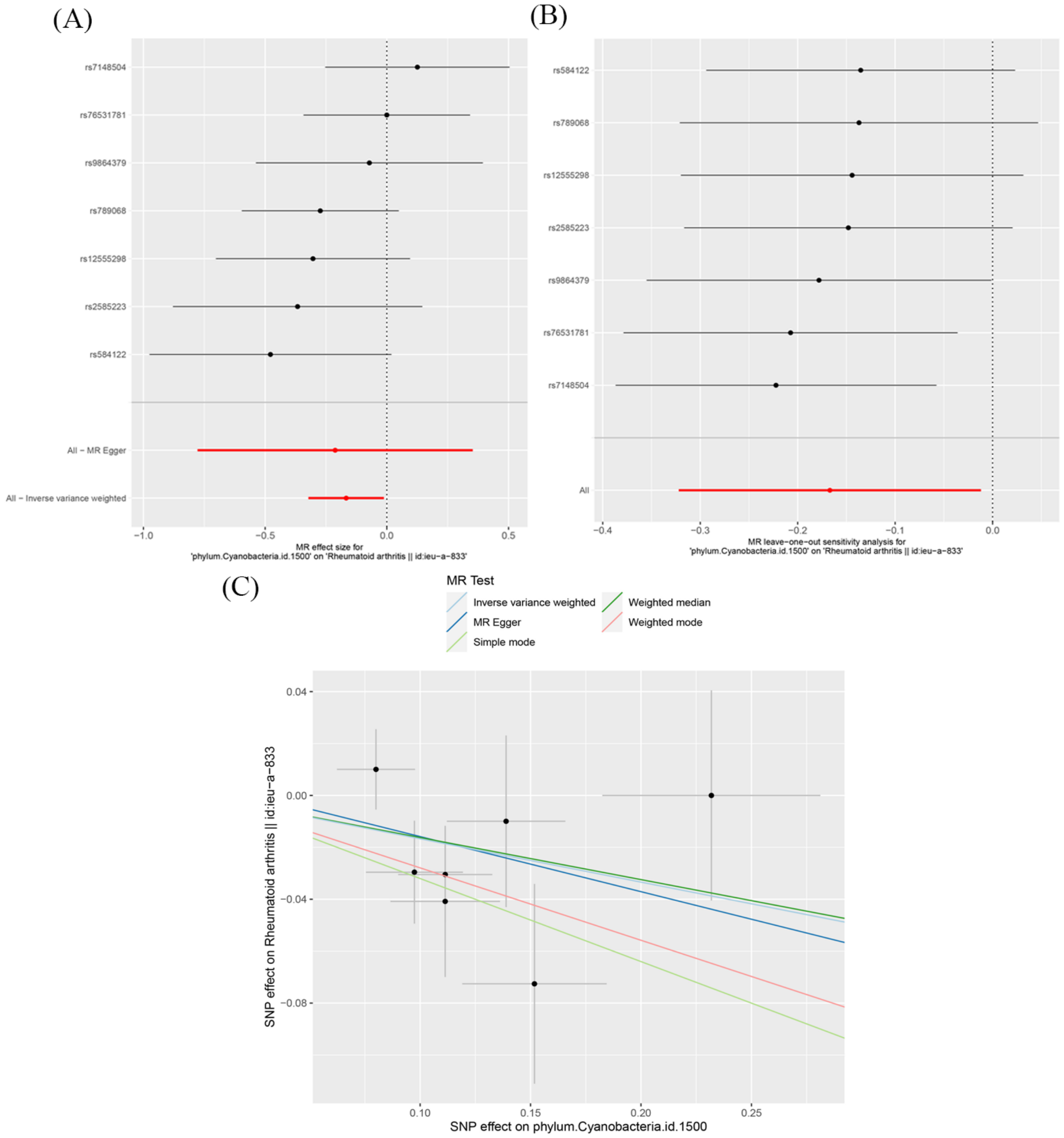

Supplementary Figure 1: (A) Forest plot, (B) Leave-one-out sensitivity analysis, and (C) Scatter plot of the causal effect of *Phylum. Cyanobacteria .id.1500* on RA risk

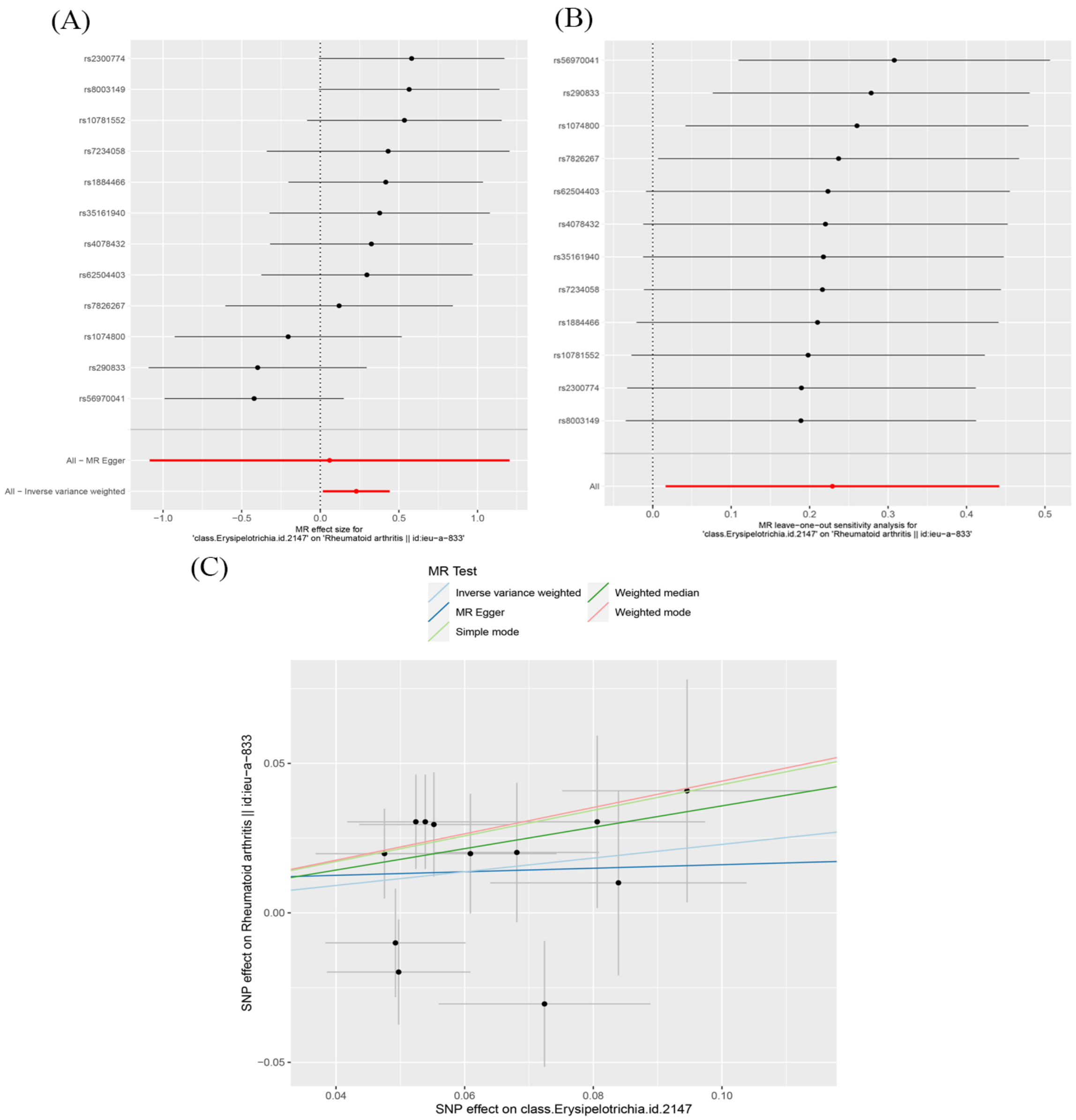

Supplementary Figure 2: (A) Forest plot, (B) Leave-one-out sensitivity analysis, and (C) Scatter plot of the causal effect of *Class Erysipelotrichia .id.2147* on RA risk

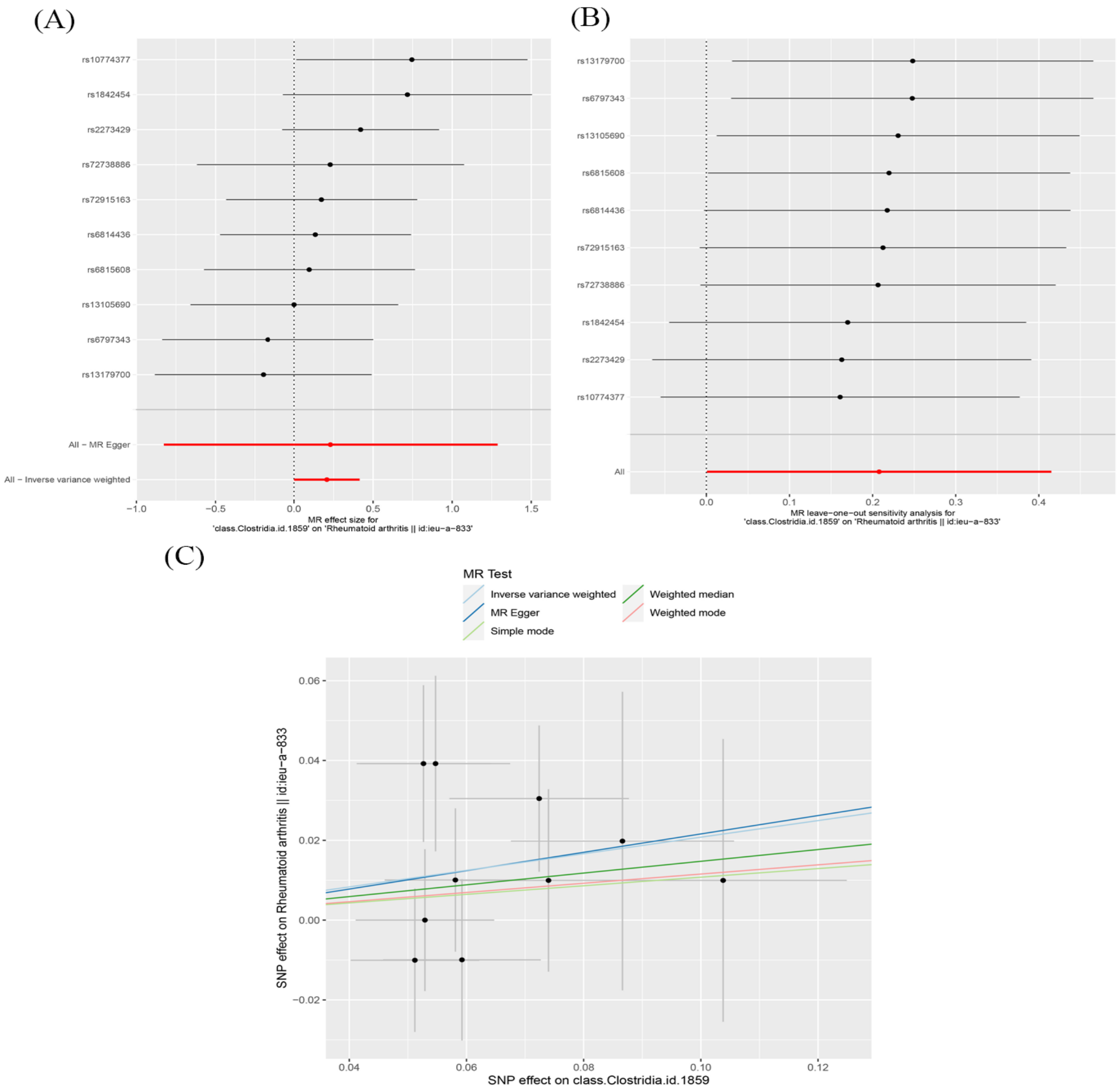

Supplementary Figure 3: (A) Forest plot, (B) Leave-one-out sensitivity analysis, and (C) Scatter plot of the causal effect of *Class.Clostridia.id.1859* on RA risk

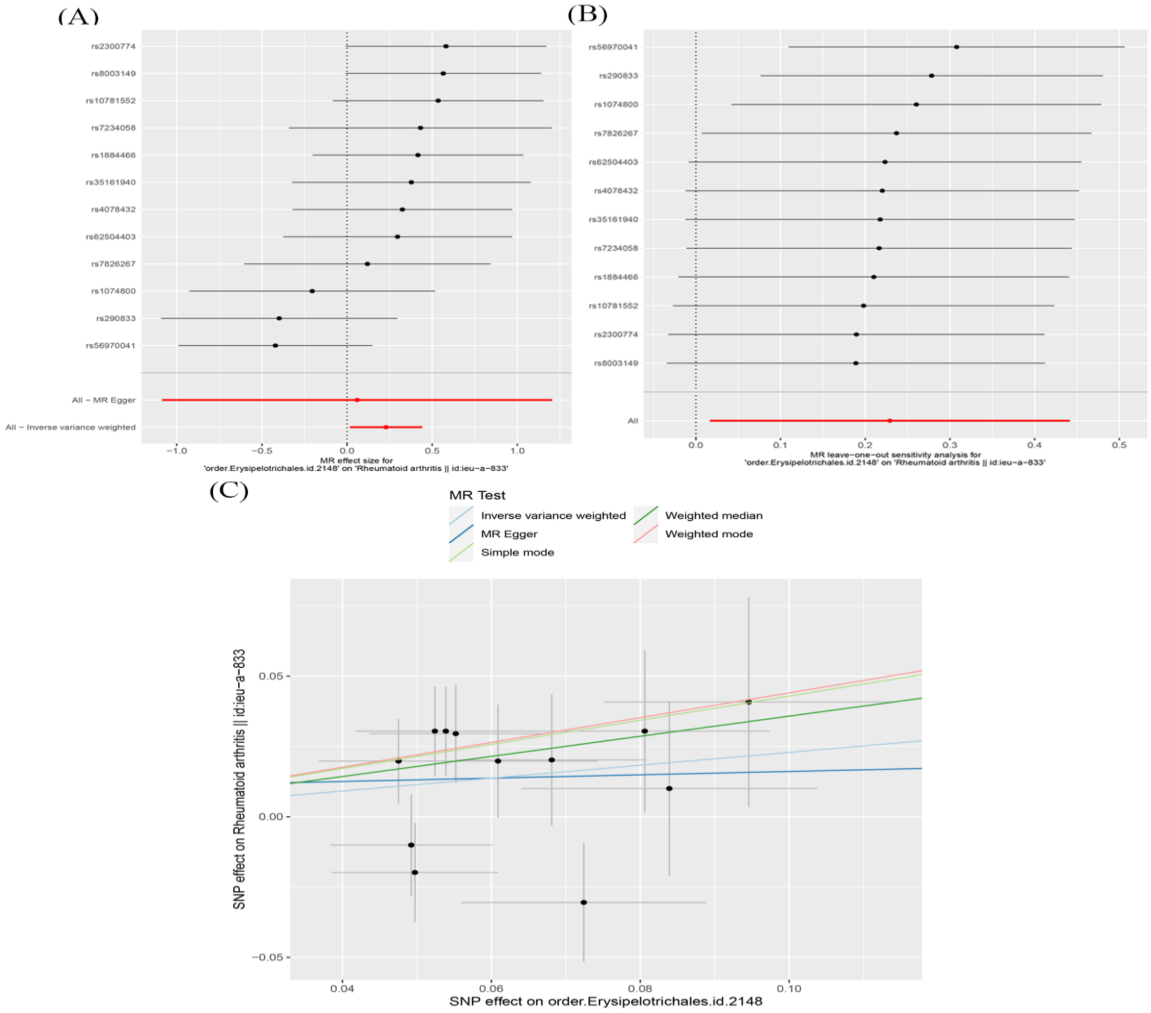

Supplementary Figure 4: (A) Forest plot, (B) Leave-one-out sensitivity analysis, and (C) Scatter plot of the causal effect of *Order.Erysipelotrichales.id.2148* on RA risk

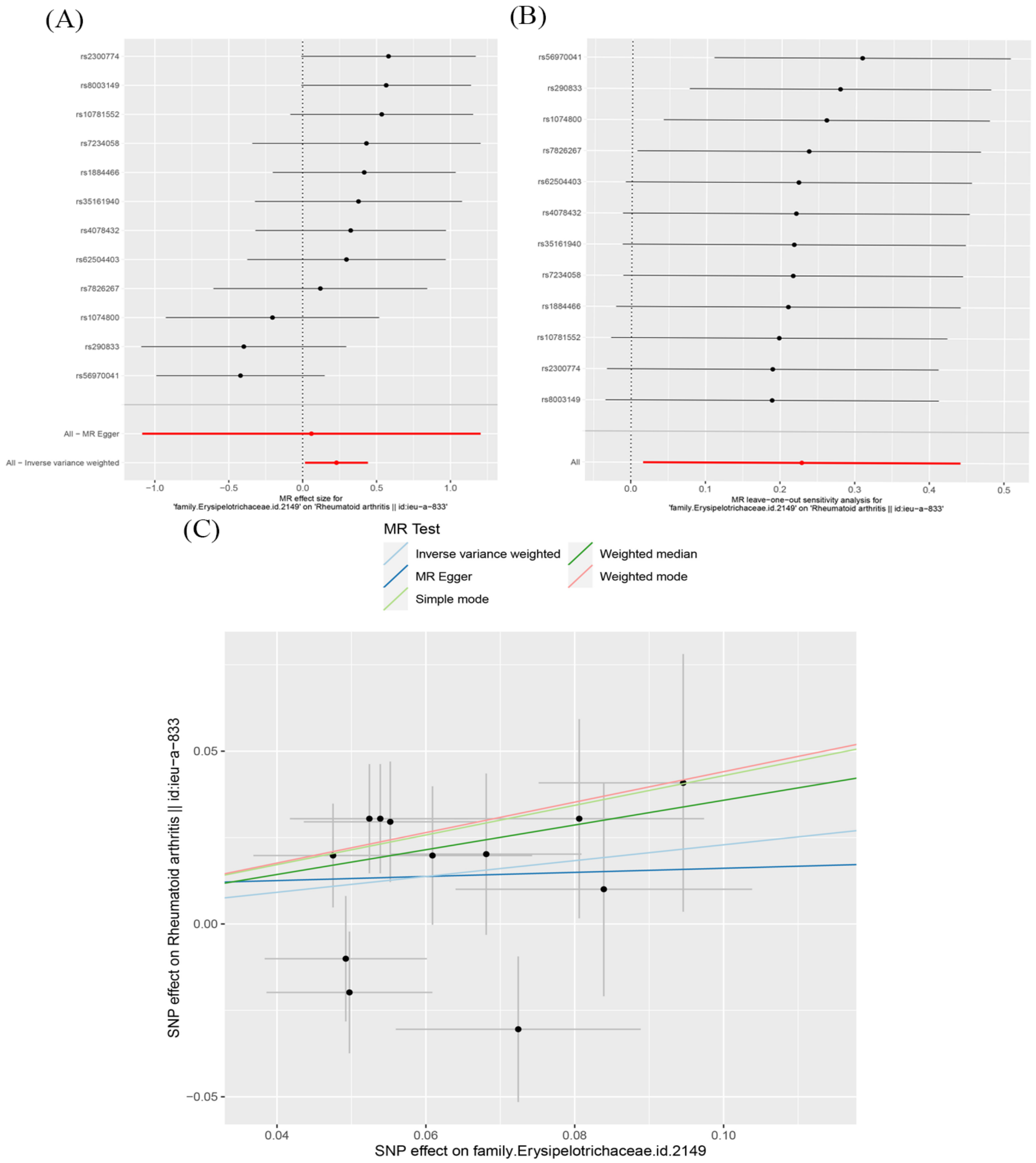

Supplementary Figure 5: (A) Forest plot, (B) Leave-one-out sensitivity analysis, and (C) Scatter plot of the causal effect of *Family.Erysipelotrichaceae.id.2149* on RA risk

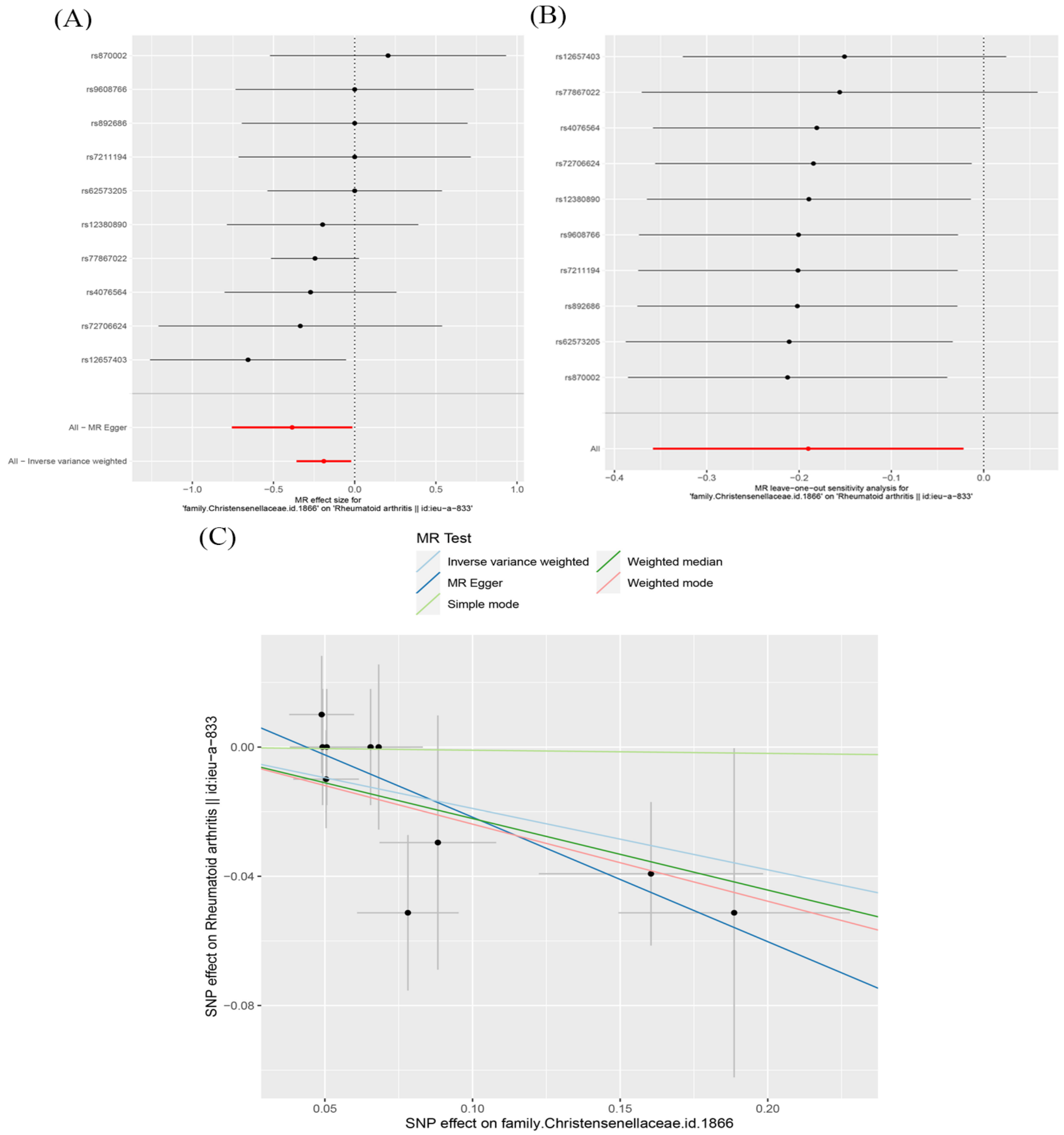

Supplementary Figure 6: (A) Forest plot, (B) Leave-one-out sensitivity analysis, and (C) Scatter plot of the causal effect of *Family.Christensenellaceae.id.1866* on RA risk

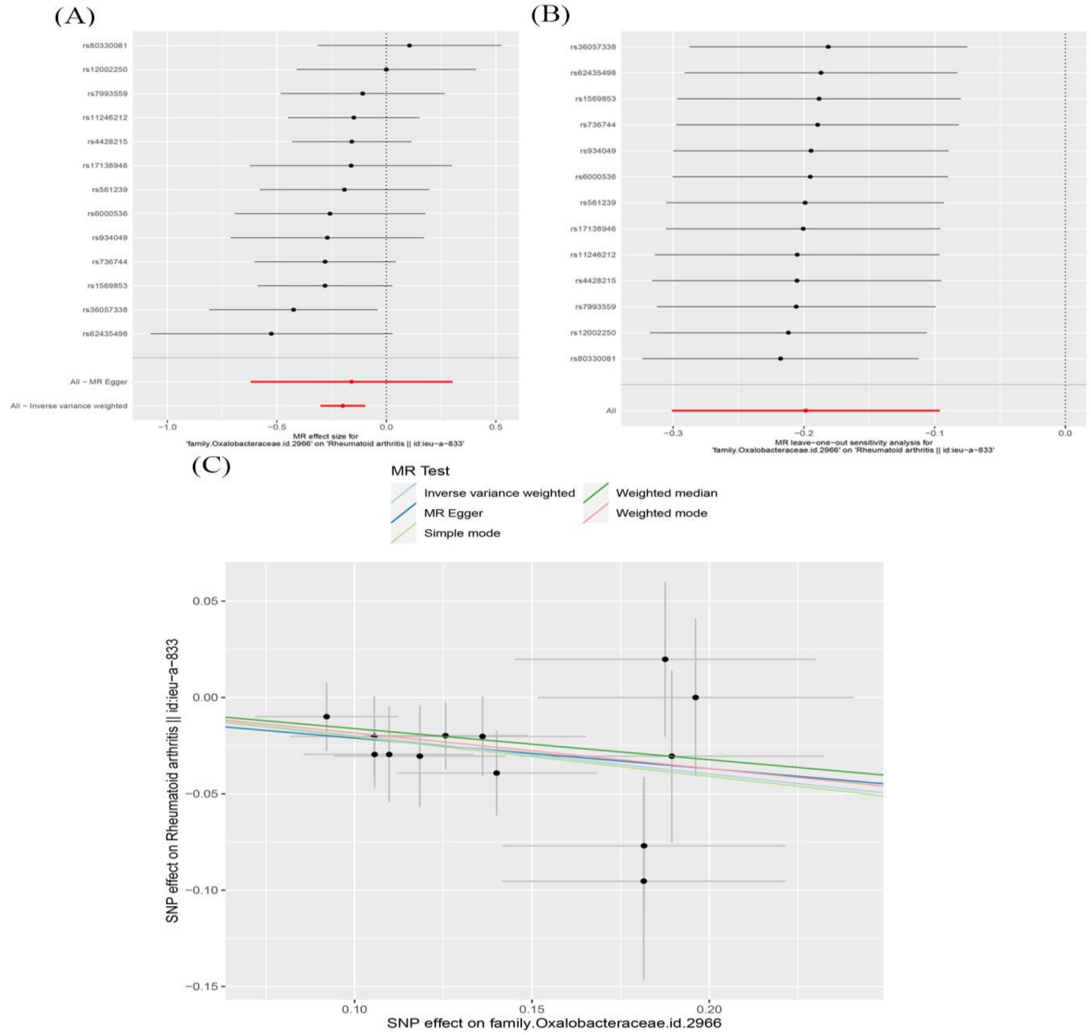

Supplementary Figure 7: (A) Forest plot, (B) Leave-one-out sensitivity analysis, and (C) Scatter plot of the causal effect of *Family.Oxalobacteraceae.id.2966* on RA risk

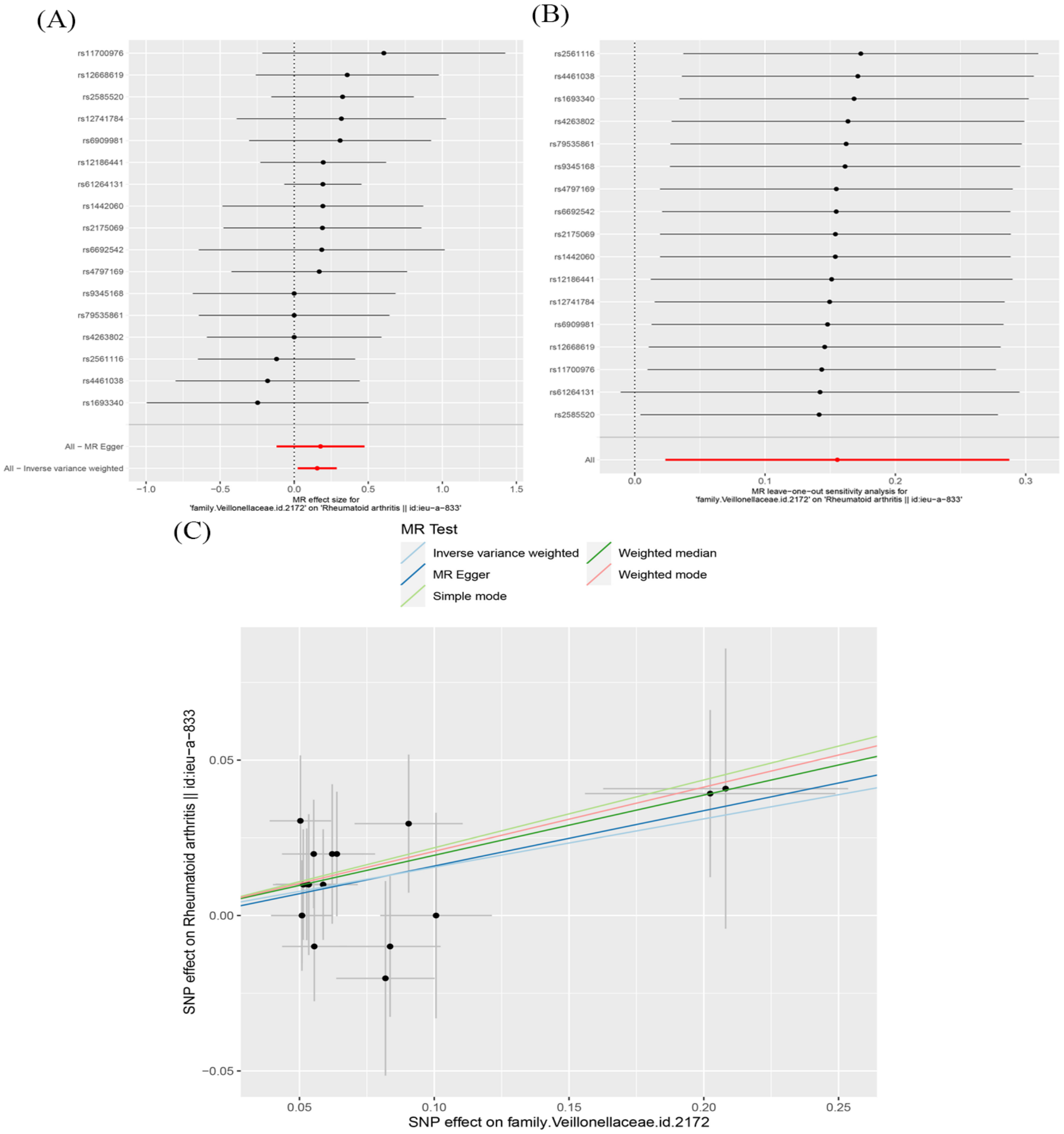

Supplementary Figure 8: (A) Forest plot, (B) Leave-one-out sensitivity analysis, and (C) Scatter plot of the causal effect of *Family.Veillonellaceae.id.2172* on RA risk

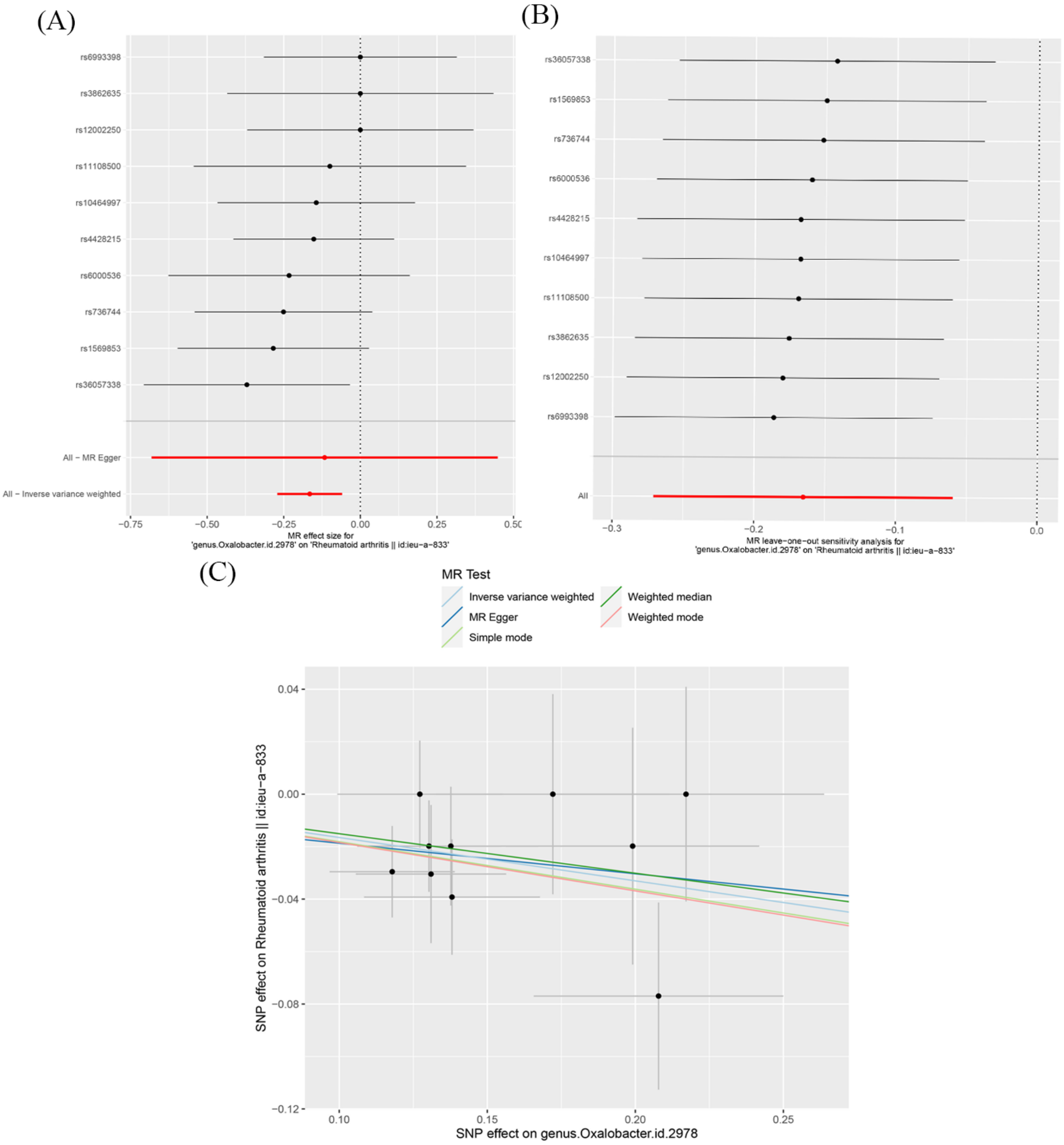

Supplementary Figure 9: (A) Forest plot, (B) Leave-one-out sensitivity analysis, and (C) Scatter plot of the causal effect of *Genus.Oxalobacter.id.2978* on RA risk

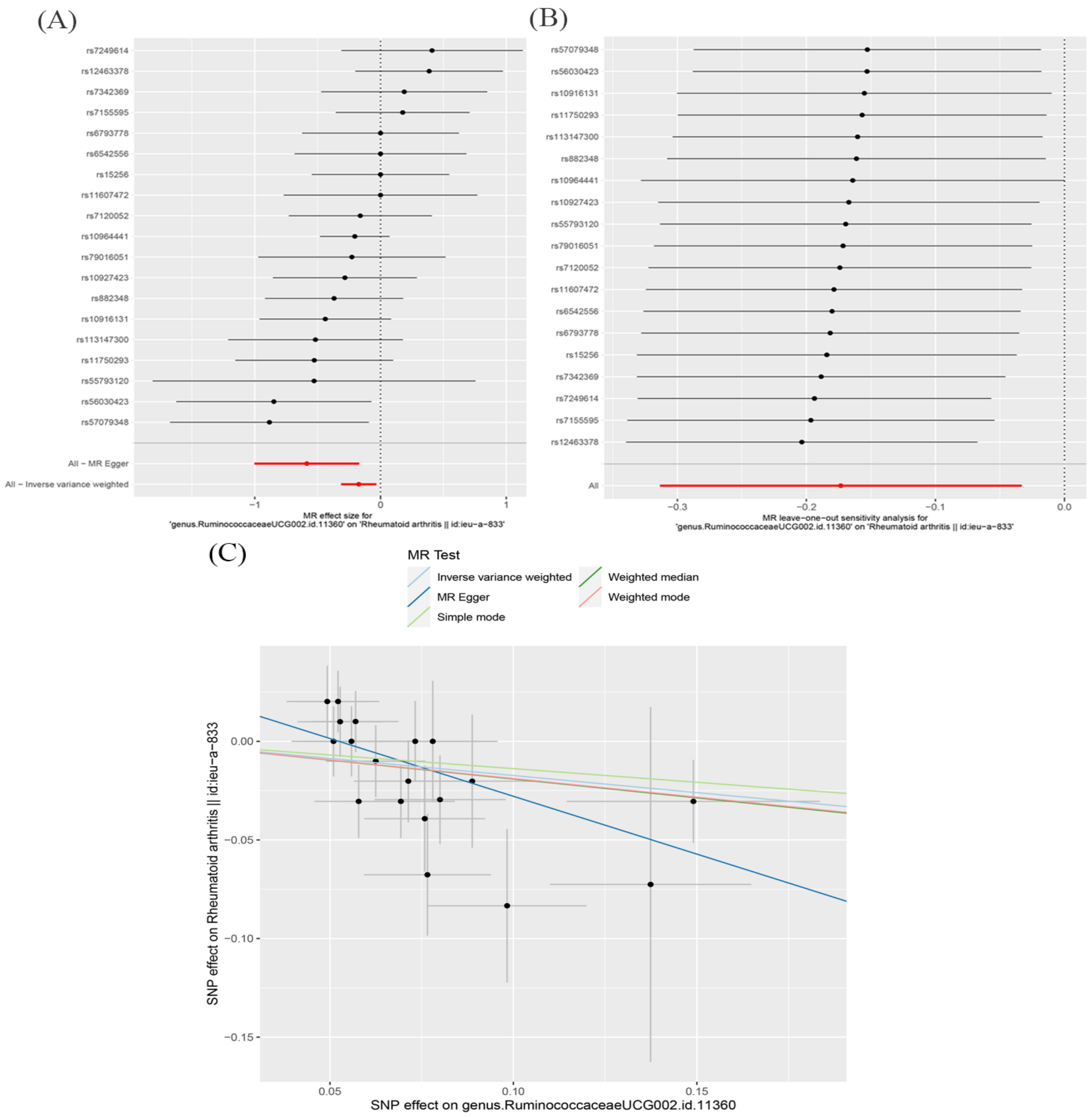

Supplementary Figure 10: (A) Forest plot, (B) Leave-one-out sensitivity analysis, and (C) Scatter plot of the causal effect of *Genus.RuminococcaceaeUCG002.id.11360* on RA risk

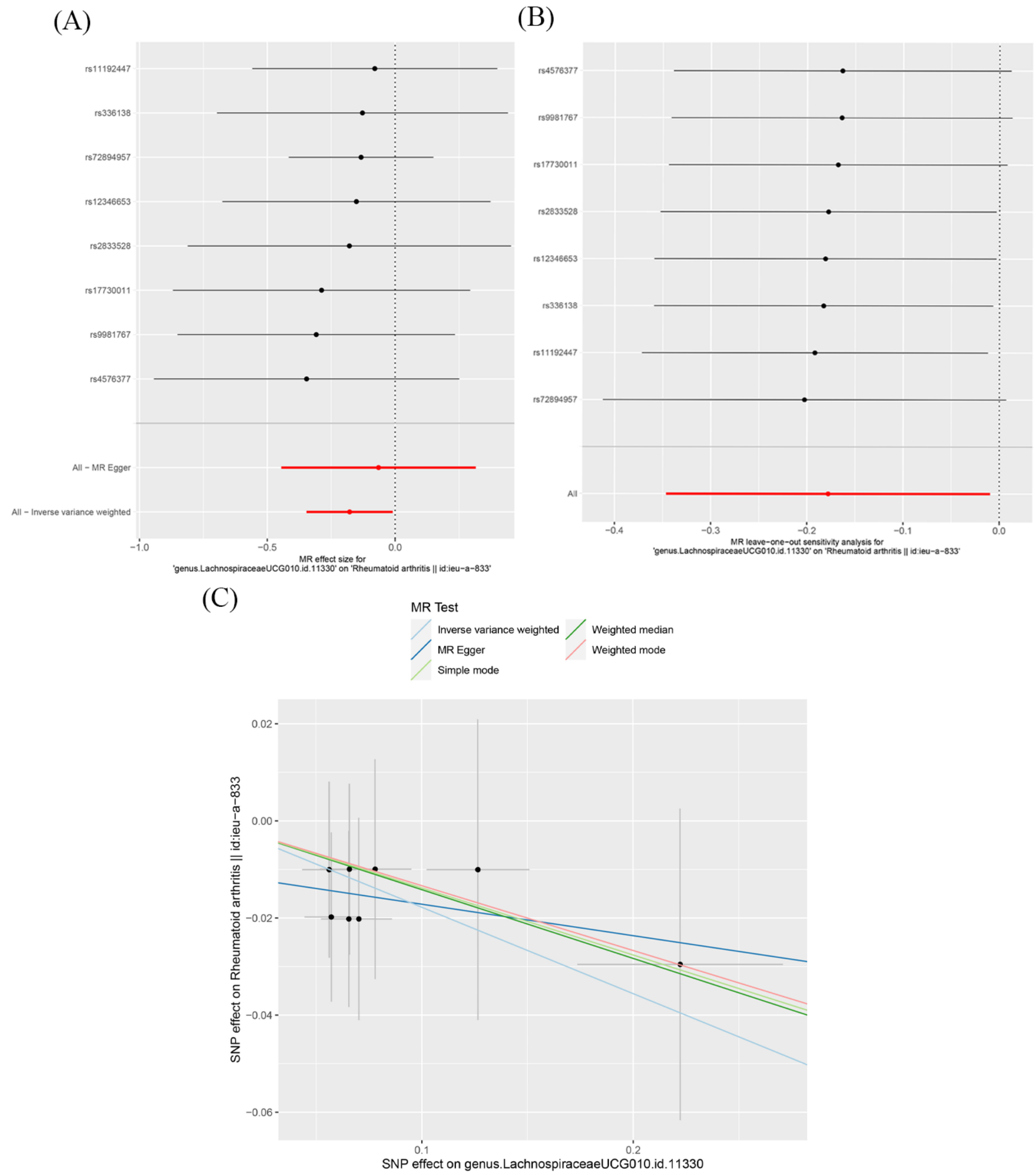

Supplementary Figure 11: (A) Forest plot, (B) Leave-one-out sensitivity analysis, and (C) Scatter plot of the causal effect of *Genus.LachnospiraceaeUCG010.id.11330* on RA risk

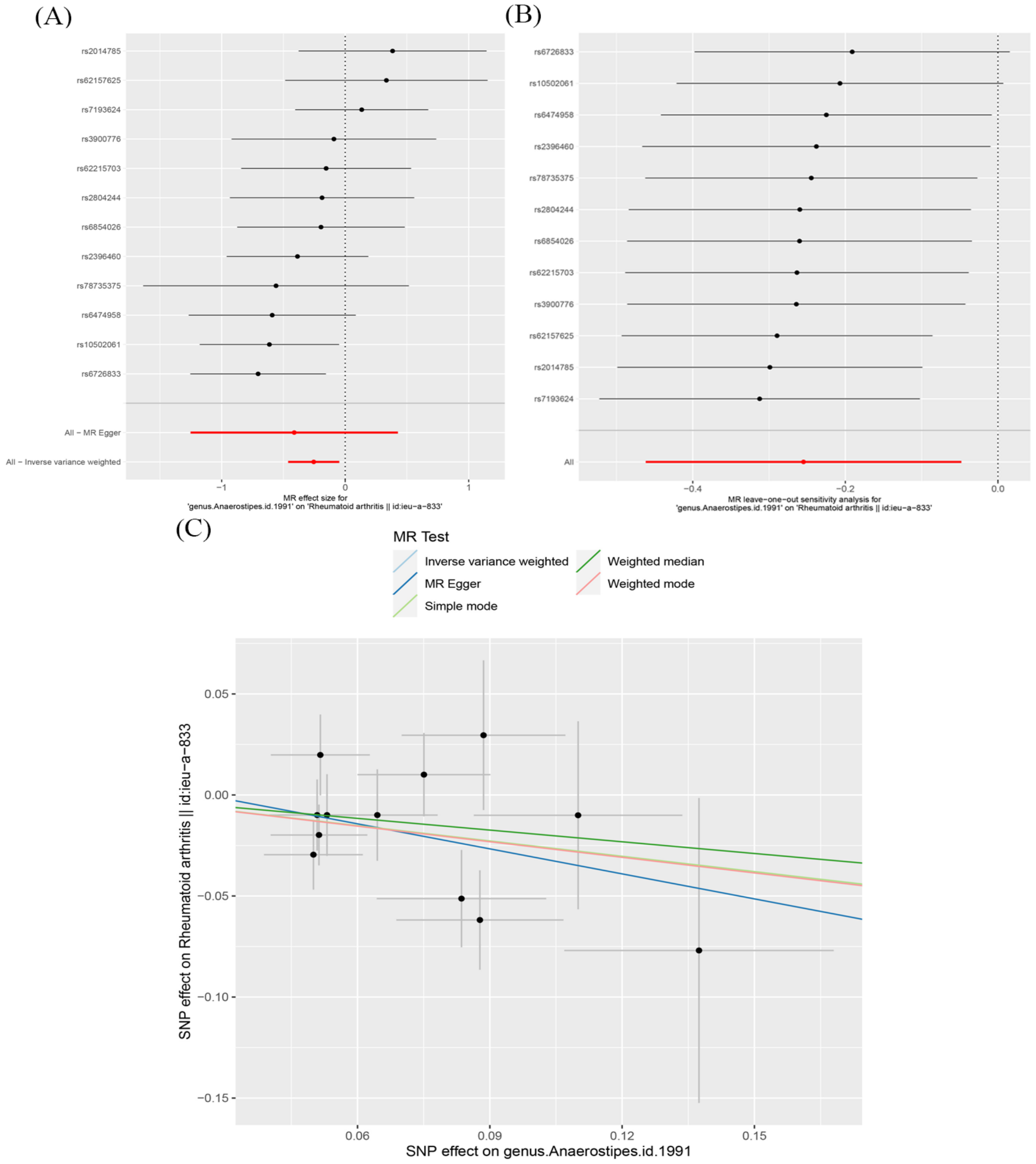

Supplementary Figure 12: (A) Forest plot, (B) Leave-one-out sensitivity analysis, and (C) Scatter plot of the causal effect of *Genus.Anaerostipes.id.1991* on RA risk

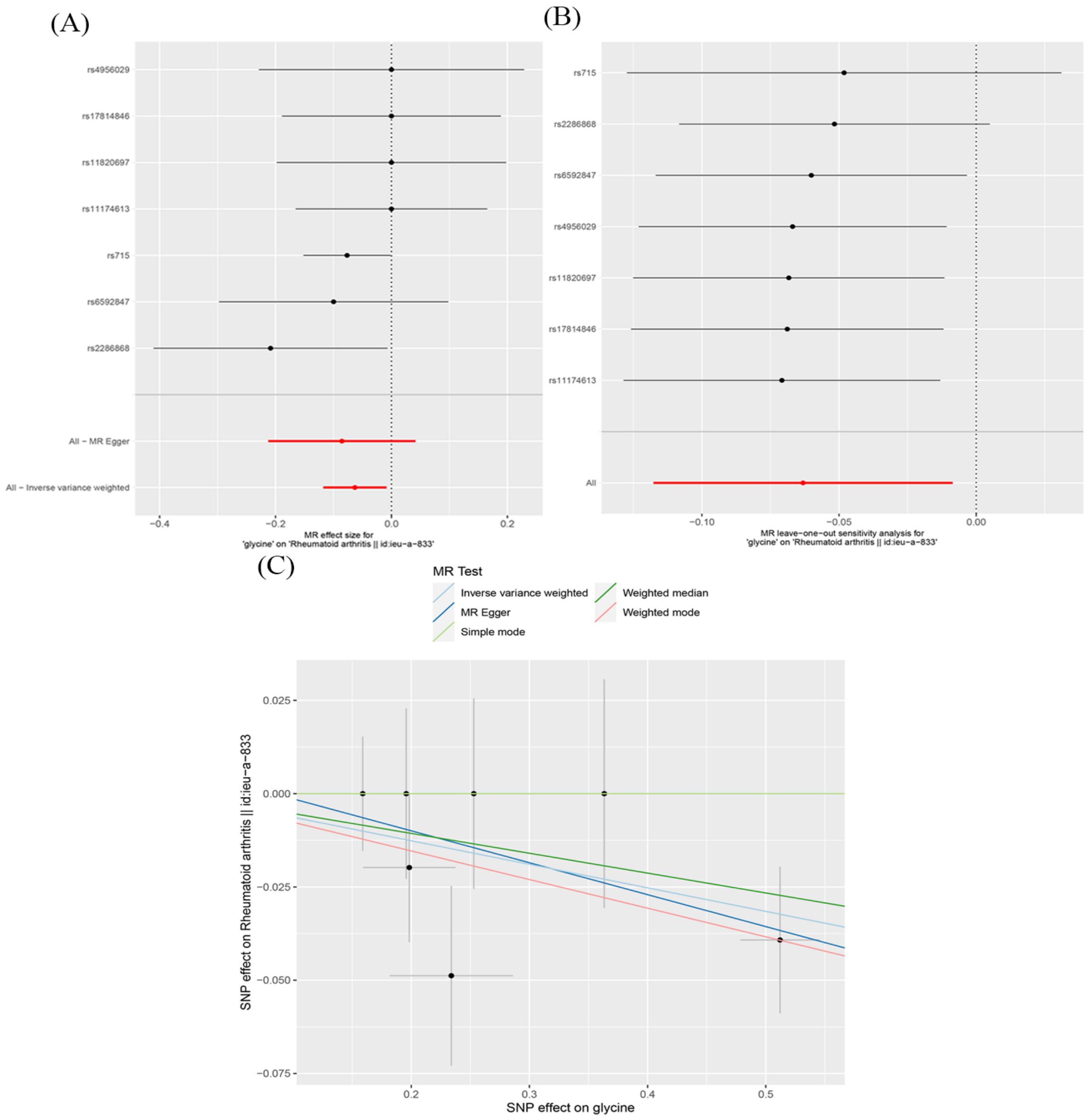

Supplementary Figure 13: (A) Forest plot, (B) Leave-one-out sensitivity analysis, and (C) Scatter plot of the causal effect of *Glycine* on RA risk

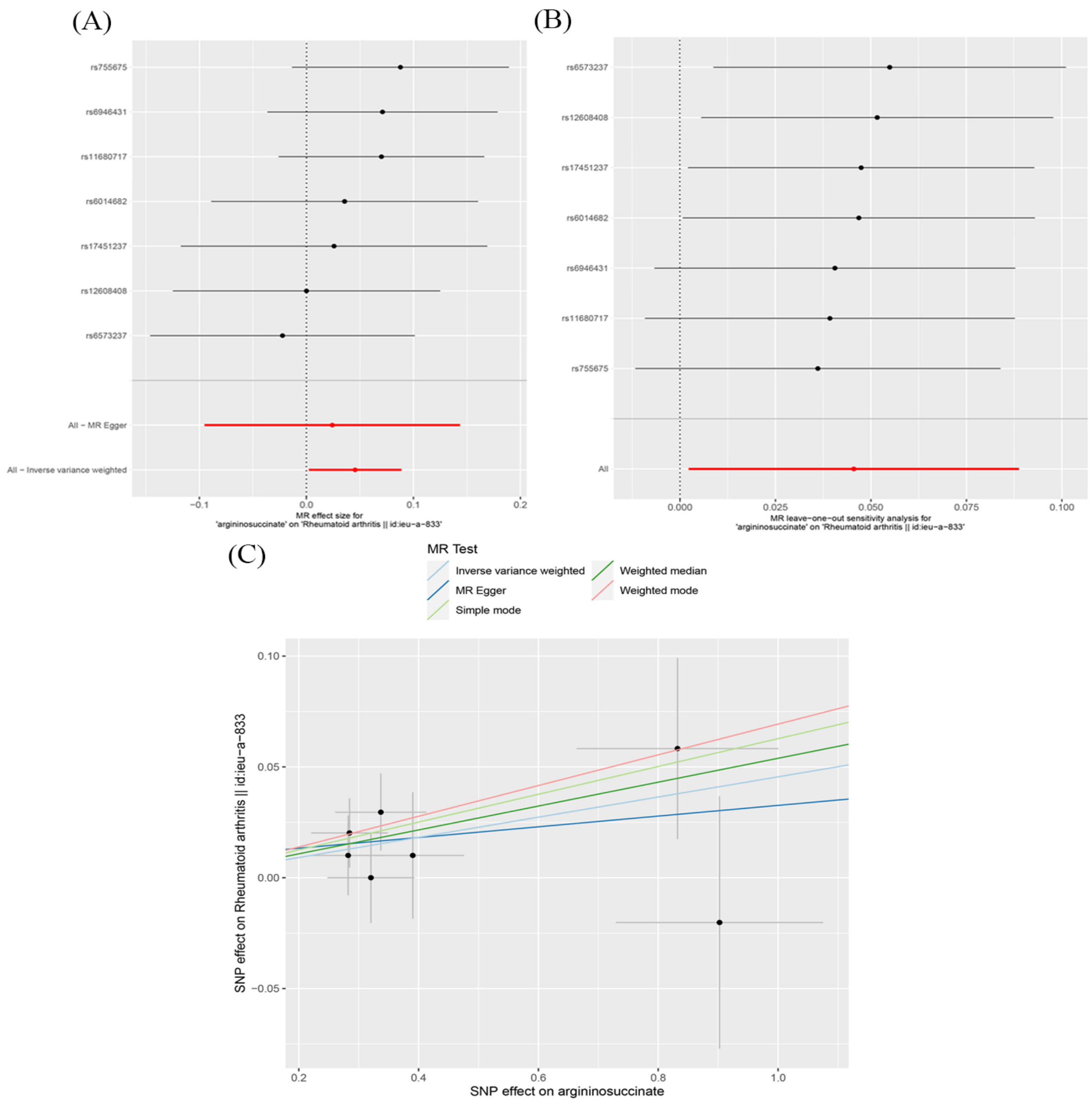

Supplementary Figure 14: (A) Forest plot, (B) Leave-one-out sensitivity analysis, and (C) Scatter plot of the causal effect of *Argininosuccinate* on RA risk

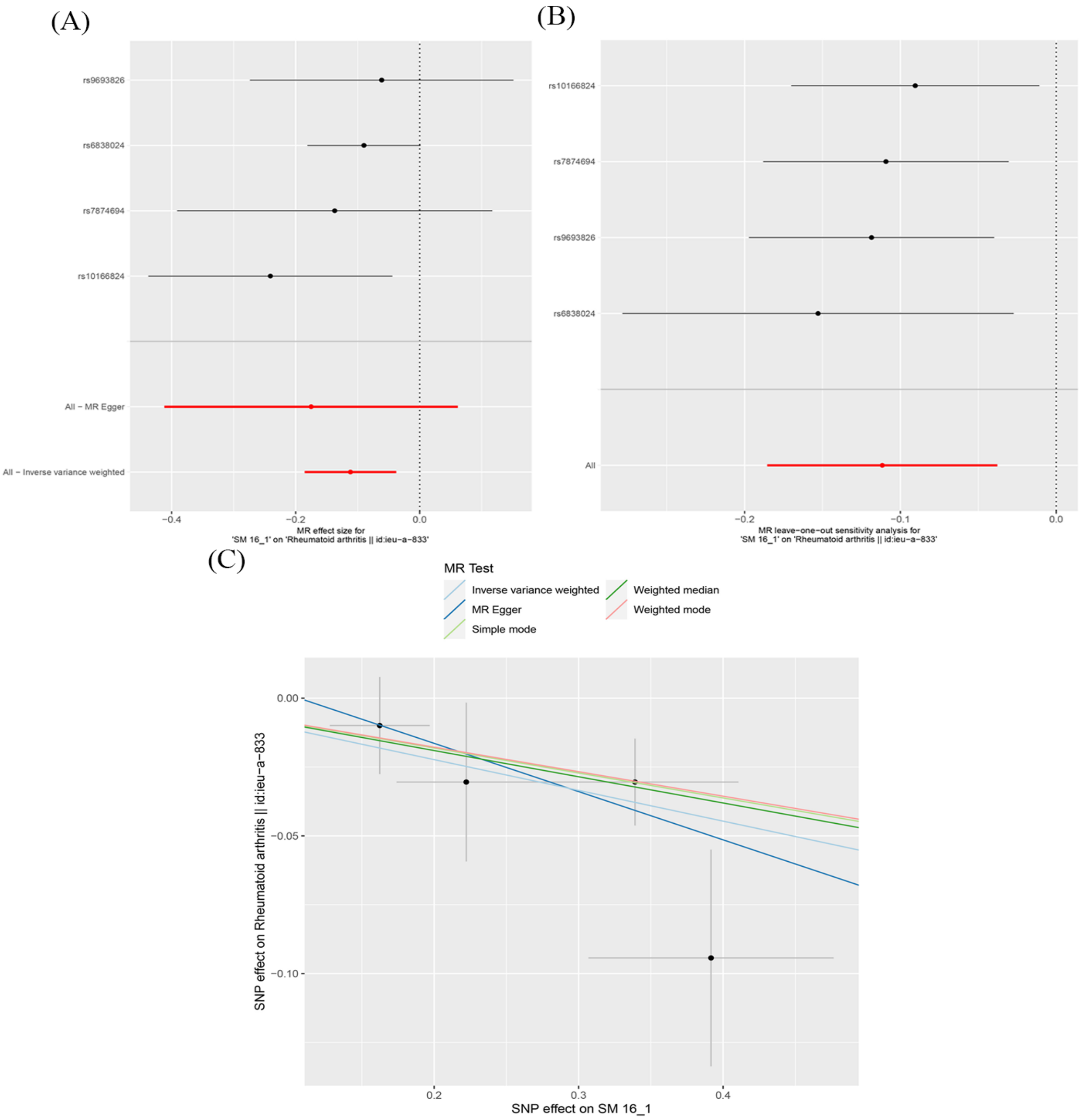

Supplementary Figure 15: (A) Forest plot, (B) Leave-one-out sensitivity analysis, and (C) Scatter plot of the causal effect of *SM 16_1* on RA risk

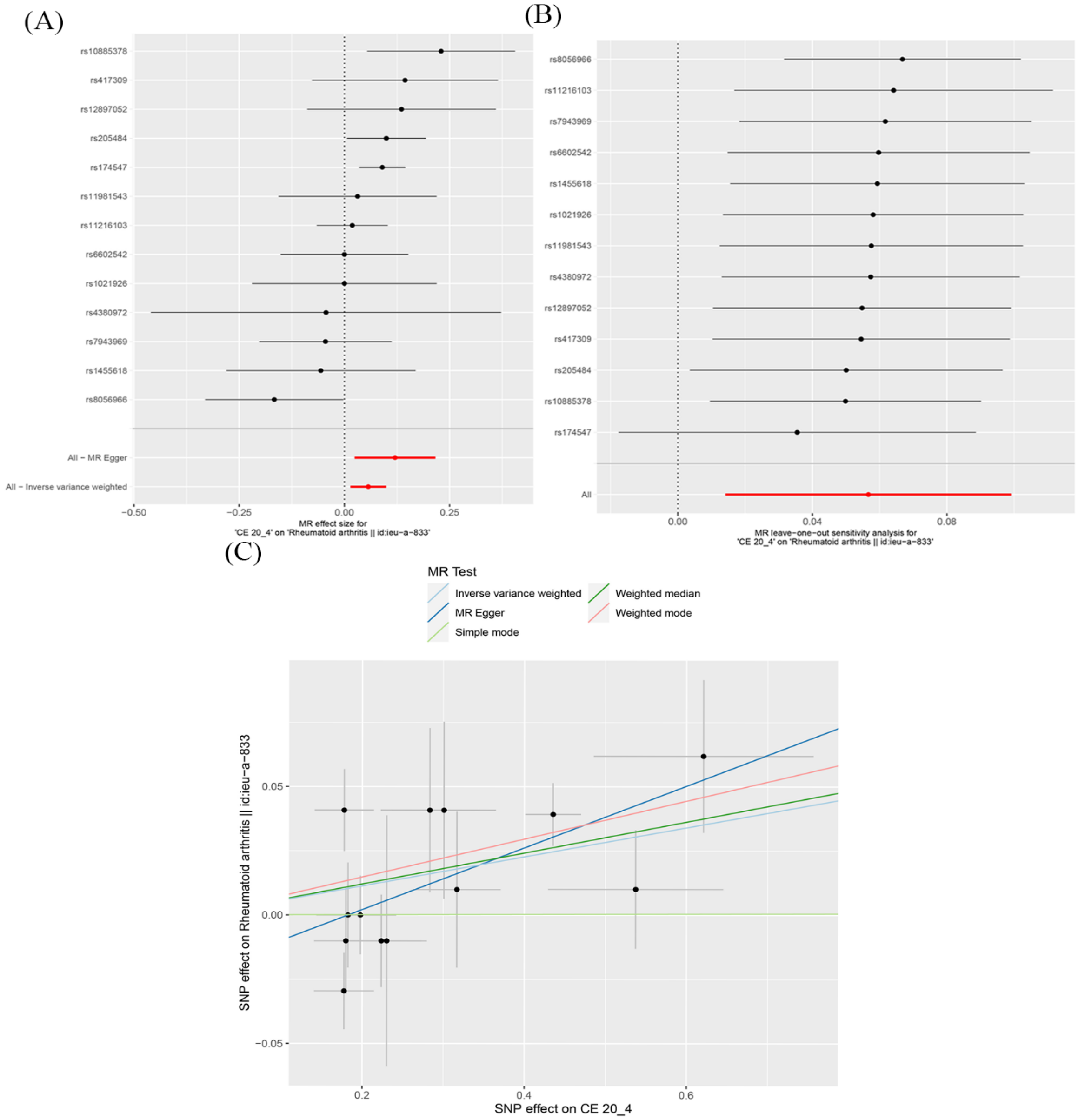

Supplementary Figure 16: (A) Forest plot, (B) Leave-one-out sensitivity analysis, and (C) Scatter plot of the causal effect of *CE 20_4* on RA risk

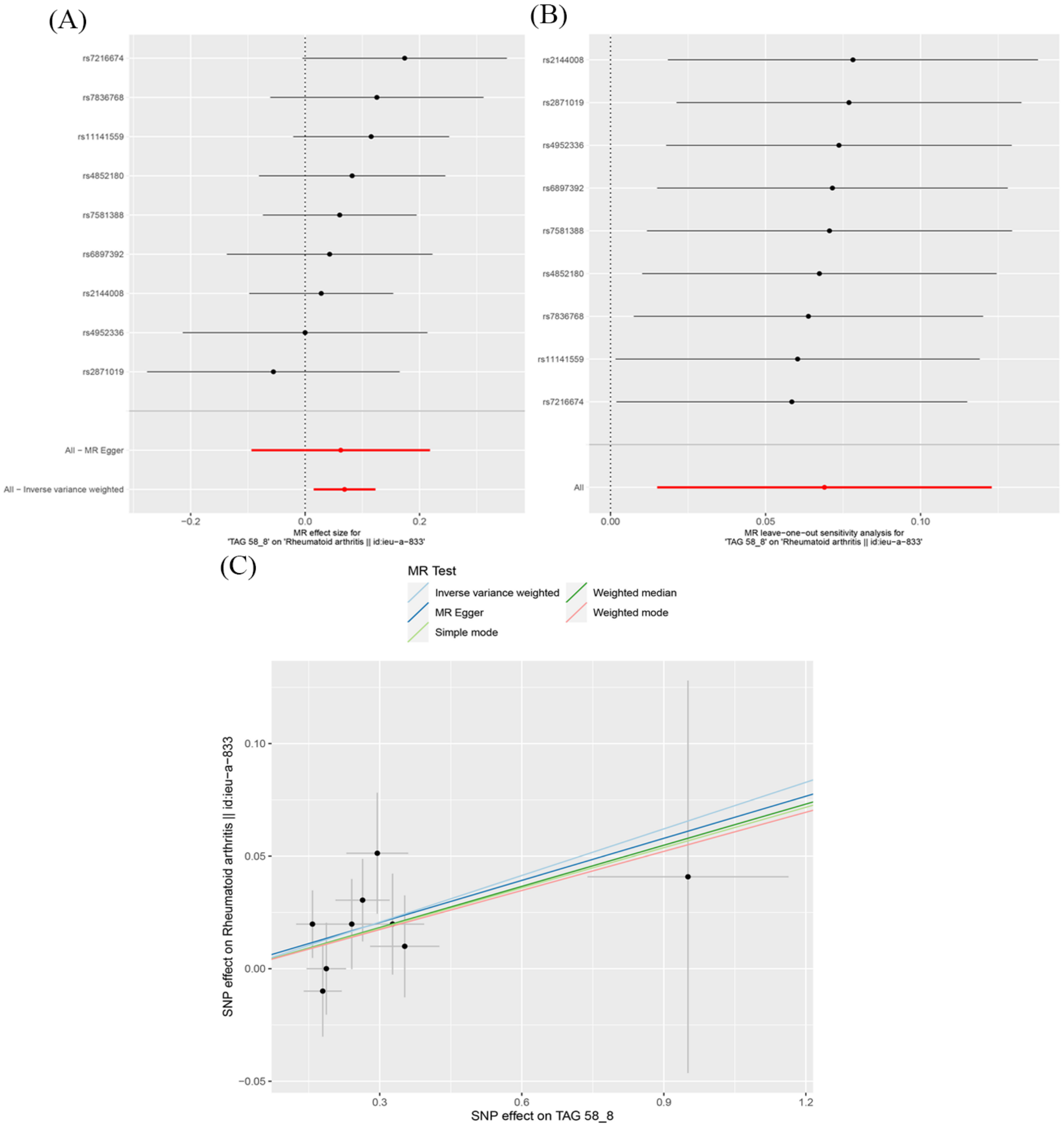

Supplementary Figure 17: (A) Forest plot, (B) Leave-one-out sensitivity analysis, and (C) Scatter plot of the causal effect of *TAG 58_8* on RA risk

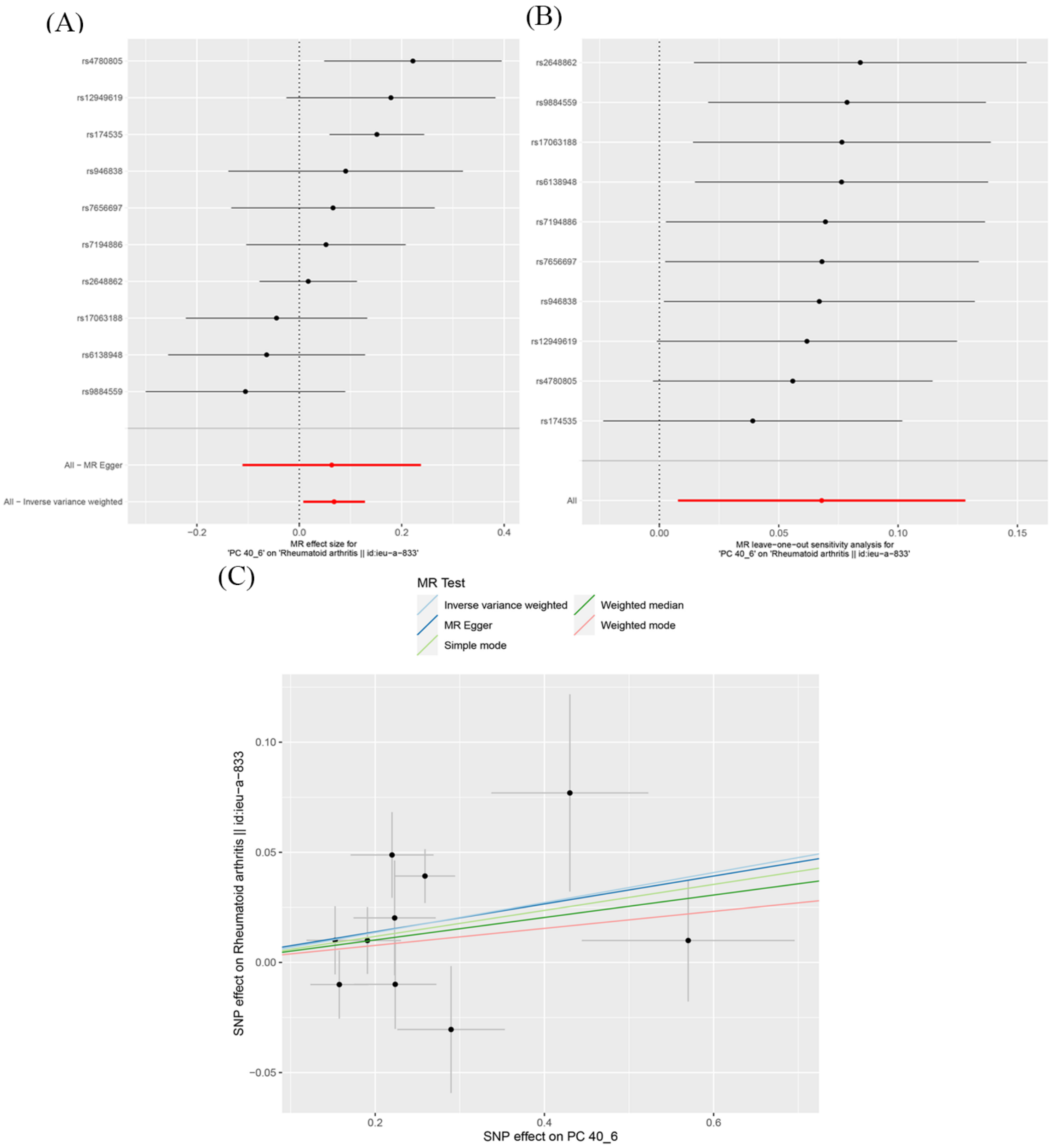

Supplementary Figure 18: (A) Forest plot, (B) Leave-one-out sensitivity analysis, and (C) Scatter plot of the causal effect of *PC 40_6* on RA risk

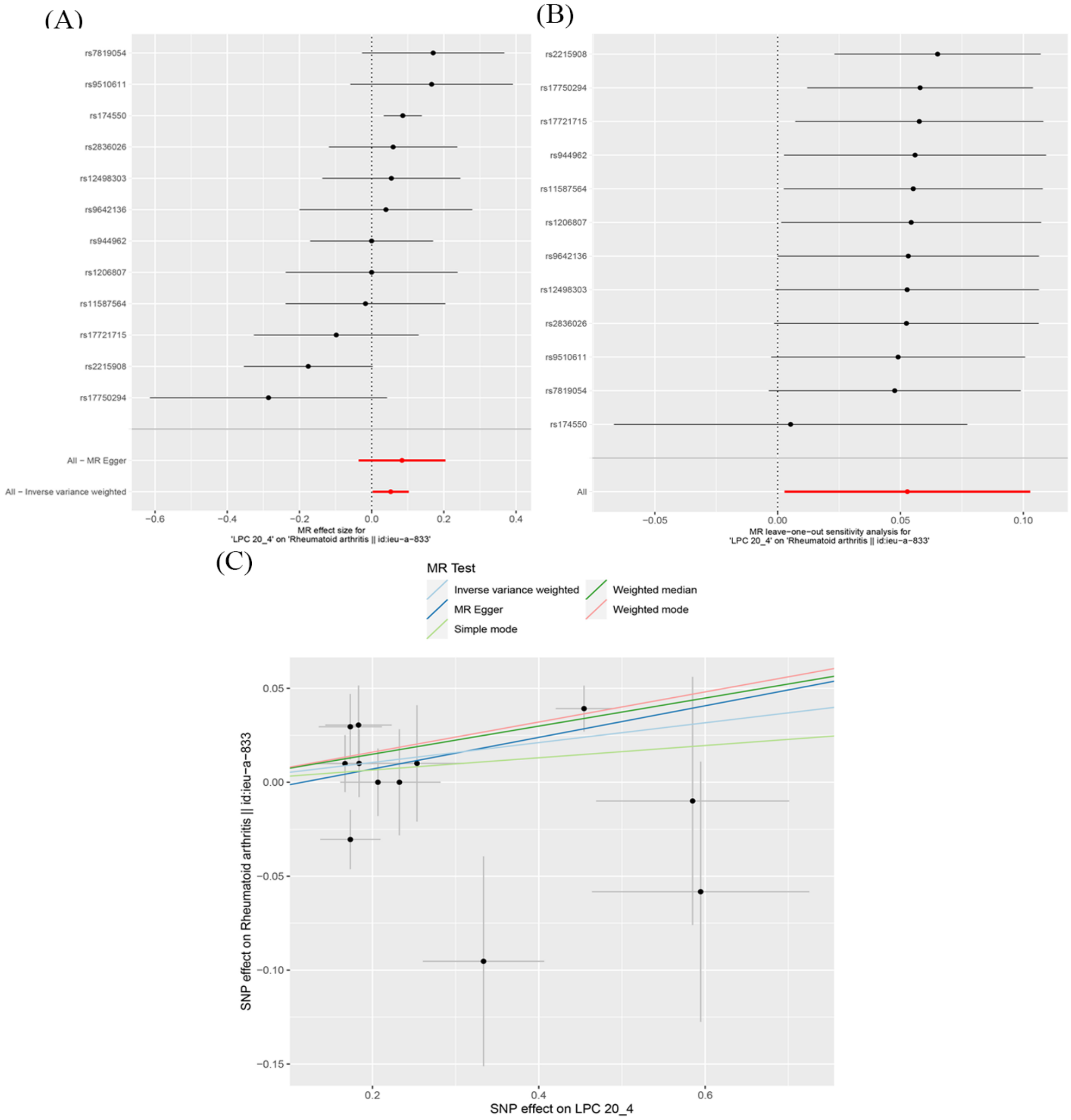

Supplementary Figure 19: (A) Forest plot, (B) Leave-one-out sensitivity analysis, and (C) Scatter plot of the causal effect of *LPC 20_4* on RA risk

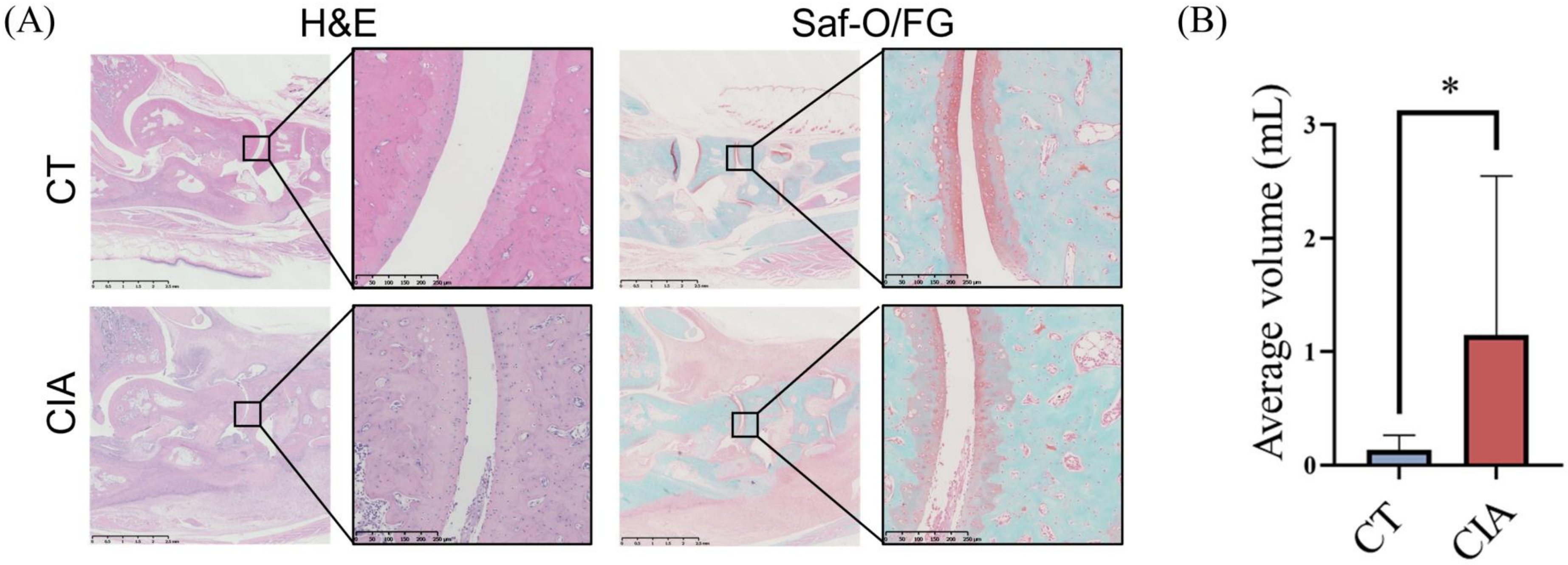

Supplementary Figure 20: (A)Representative HE staining of ankle joints in rats; (B) Average alterations of hind paws' volumes. H＆E, hematoxylin-eosin staining; Saf-O/FG, Safranin O-Fast Green staining; CT, control group; CIA, collagen-induced arthritis group. *$p < 0.05$.
